# How the mechanobiology orchestrates the iterative and reciprocal ECM-cell cross-talk that drives microtissue growth

**DOI:** 10.1126/sciadv.add9275

**Published:** 2023-03-29

**Authors:** Mario C. Benn, Simon A. Pot, Jens Moeller, Tadahiro Yamashita, Charlotte M. Fonta, Gertraud Orend, Philip Kollmannsberger, Viola Vogel

**Affiliations:** ^1^ Laboratory of Applied Mechanobiology, Department of Health Sciences and Technology, ETH Zurich, Vladimir-Prelog-Weg 4, Zurich 8093, Switzerland.; ^2^ The Tumor Microenvironment Laboratory, INSERM U1109, Hôpital Civil, Institut d'Hématologie et d'Immunologie, 1 Place de l'Hôpital, Strasbourg 67091, France.; ^3^ Université Strasbourg, Strasbourg 67000, France.; ^4^ Fédération de Médecine Translationnelle de Strasbourg (FMTS), Strasbourg 67000, France.; ^5^ Biomedical Physics, Heinrich-Heine-University Düsseldorf, Universitätsstrasse 1, Düsseldorf 40225, Germany.

## Abstract

Controlled tissue growth is essential for multicellular life and requires tight spatiotemporal control over cell proliferation and differentiation until reaching homeostasis. As cells synthesize and remodel extracellular matrix, tissue growth processes can only be understood if the reciprocal feedback between cells and their environment is revealed. Using de novo–grown microtissues, we identified crucial actors of the mechanoregulated events, which iteratively orchestrate a sharp transition from tissue growth to maturation, requiring a myofibroblast-to-fibroblast transition. Cellular decision-making occurs when fibronectin fiber tension switches from highly stretched to relaxed, and it requires the transiently up-regulated appearance of tenascin-C and tissue transglutaminase, matrix metalloprotease activity, as well as a switch from α5β1 to α2β1 integrin engagement and epidermal growth factor receptor signaling. As myofibroblasts are associated with wound healing and inflammatory or fibrotic diseases, crucial knowledge needed to advance regenerative strategies or to counter fibrosis and cancer progression has been gained.

## Teaser

Cellular mechanobiology reshapes the extracellular matrix, and their iterative interplay drives tissue growth and maturation.

## INTRODUCTION

Controlled tissue growth and maturation processes to gain homeostasis, as well as the rapid tissue repair upon injury, are essential for the emergence of multicellular life and are elaborately coordinated in space and time like a well-orchestrated symphony. Tissue growth and remodeling processes are tightly linked to the production, assembly, and remodeling of extracellular matrix (ECM) and to the transiently up-regulated appearance of other proteins (*1*–*3*). During tissue growth, an initial fibronectin (Fn)–rich ECM is assembled (*2*, *3*), which templates the subsequent deposition of collagen fibers as a tension-bearing element in the maturing tissue (*4*, *5*). Tissue maturation shifts the tensional load from active cell contractility toward passive ECM tension, thereby enhancing the mechanical stability of the emerging tissue (*1*, *6*). This is particularly relevant during embryonic development, postnatal growth, and tissue regeneration after injury, where maturation processes are visible as steep gradients in the composition and organization of cell phenotypes and ECM, often at the length scale of single cells (*7*). ECM production, cell proliferation, contractile forces, and subsequent tissue contractions are thus central elements of tissue growth, maturation, and repair (*8*). Tissue repair is particularly time sensitive, as the reestablishment of tissue integrity and tension is essential for wound closure and, therefore, crucial for survival (*9*). The failure to temporally coordinate central sequential tissue repair steps is known to lead to scarring and major impairment of tissue functions, often by initiating fibrotic pathologies, potentially even leading to organ failure and death of the organism (*10*).

Fibroblasts are abundant cells residing in the stroma of nearly all mammalian organ tissues and are crucial for ECM secretion, assembly, and remodeling (*11*). As such, fibroblasts play major roles in tissue growth and repair. Upon injury, growth factors released at wound sites [most notably transforming growth factor–β1 (TGF-β1) and platelet-derived growth factor], the influence of certain wound bed ECM components [e.g., extra domain A (ED-A) Fn], and alterations of wound mechanics (e.g., tissue tension and ECM fiber tension) induce the activation of resident tissue fibroblasts, thus transitioning into activated myofibroblasts by mechanisms that are not fully understood (*11*). Myofibroblasts are characterized by the expression of alpha smooth muscle actin (αSMA), which leads to an increased contractility and cytoskeletal stress fiber formation, and by enhanced ECM production (*8*, *11*, *12*). In early embryogenesis or upon injury, Fn is the first structural protein that is assembled by activated fibroblasts and myofibroblasts and, therefore, an important component of the provisional ECM (*2*). Fn contains a large number of molecular binding sites for proteins and cells, providing both biochemical and mechanical cues for adherent cells (*2*, *13*). Through partial protein unfolding, mechanical tension–induced Fn fiber stretching can either cause the deactivation of exposed binding sites or the exposure of otherwise cryptic binding sites and, thus, switch Fn’s binding affinity to matrix proteins or alter the bioavailability of cellular binding sites (*14*). For example, unstretched Fn fibers are the preferential ECM template for collagen I fiber assembly (*5*), whereas Fn fibril formation, bundling, and ECM assembly are accelerated by the exposure of cryptic Fn-Fn self-assembly sites in stretched Fn fibers due to applied cell tension (*14*). In addition, partial Fn unfolding by fiber stretching was proposed to expose the cryptic Toll-like receptor 4 (TLR-4)–activating epitope on ED-A Fn, a Fn splice variant associated with fibrosis and known to drive myofibroblast transition and collagen gene expression via TLR-4–dependent signaling (*14*–*16*). As such, Fn fiber stretch assays revealed that the Fn fiber strain and, thus, the enhanced presence of partially exposed Fn domains directly regulate ECM assembly and the fate of adhering cells (*5*, *14*, *17*). How Fn fiber tension and its mechanoregulated exposure of binding sites influence the transition toward a more mature collagen-rich ECM and how this process enables the concomitant transition of myofibroblasts to fibroblasts during tissue growth and repair are unclear.

In response to enhanced cellular traction forces, ECM fibers get aligned and stretched, concomitant to the alteration of protein expression programs, which leads to further ECM production and remodeling. In an iterative process, the remodeled ECM triggers a next round of adhesion assembly, integrin signaling, cytoskeletal remodeling, and traction force generation, among others, which again induces alterations in gene transcription, thereby illustrating the powerful concept of iterative and time-dependent cell-ECM reciprocity. Under pathological conditions, such as inflammatory and fibrotic diseases, or cancer, myofibroblasts remain constitutively active for extended time periods (*11*, *12*).

The up-regulated contractile forces that myofibroblasts apply to the surrounding ECM fibers finally drive wound closure (*8*). These forces are transmitted to the ECM via integrins. Integrins are mechanosensory units that couple the ECM to the contractile cytoskeleton (*18*). Tensile forces applied to integrins lead to rapid maturation and strengthening of cell-ECM adhesion complexes (*18*). The downstream signaling of integrins is known to play a central role in the regulation of αSMA expression in myofibroblasts (*19*). Integrin signaling is therefore central to reciprocal cell-ECM interactions and depends on the composition of the ECM as different integrin heterodimers are exploited by cells to specifically recognize distinct ECM components (*20*). Beyond the structural ECM proteins, including Fn and collagens, which are linked via their respective integrins to the contractile cytoskeleton, a range of matrisomal proteins exists too. These matrisomal proteins have regulatory roles and are transiently expressed during tissue growth and repair. Therefore, they are sometimes used as time-specific biomarkers of tissue health or disease (*3*, *7*, *21*). Two examples are tenascin-C (TNC) and tissue transglutaminase (TG2), both of which are quickly up-regulated at sites of ECM remodeling (*3*, *22*). TNC is a large multivalent hexameric matricellular glycoprotein, which plays multiple roles in the early and late phases of wound healing (*23*), and is critical for tissue stability (*24*–*27*). The main function of TG2 is to cross-link ECM proteins with varying targets among ECM fibrils, including Fn, fibrillin, and several collagen types (*28*), thus stiffening the ECM and protecting the ECM from proteolytic degradation (*29*). In diseased ECM, enhanced cross-linking is typically associated with reduced ECM compliance (*30*). Thus, targeting mechanisms that lead to a pathological increase of ECM stiffness was proposed to advance treatment strategies (*31*, *32*). Although TG2 and lysyl oxidase (LOX) represent the most prominent ECM cross-linkers, recent reports indicate a higher clinical relevance for TG2 than LOX (*33*). By virtue of its targets, TG2 is an important stabilizer of both the early and more mature wound healing matrix, while LOX was reported to be mostly active after full assembly of the collagen matrix at later stages of tissue maturation (*22*). Both TNC and TG2 are up-regulated in regions of mechanical stress (*34*, *35*), as well as during embryonic development, wound healing, and in fibrotic or neoplastic pathologies (*36*–*39*). Like TNC (*40*), TG2 is a known myofibroblast activator (*38*). It remains unknown how the spatiotemporal distribution of TNC and TG2 and their physical and enzymatic cross-linking activities affect tissue maturation and the fibroblast-to-myofibroblast phenotype transition (F-M-T) or its reversal. Whether a functional interplay between TNC and TG2 exists remains unknown as well.

As many biophysical features of three-dimensional (3D) tissues are poorly recapitulated by the reduced dimensionality of 2D cell culture approaches (*12*, *13*, *41*), many aspects of how biochemical and biomechanical signaling cooperate to regulate tissue growth and maturation processes remain elusive (*14*). While de novo–grown organoids have been powerful model systems, they have their limitations due to substantial organoid-to-organoid variability (*42*), which makes it more difficult to systematically tune parameters of interest and quantify their impacts. As the importance of the reciprocity between the ECM and cells is increasingly recognized, other in vitro methods were developed for the production of 3D microtissues (μTissues) using, for example, punched-out macroporous collagen scaffolds that allow investigation of early and late stages of tissue maturation (*9*). However, most of the current 3D μTissue platforms lack the potential to translate spatial to temporal information, or are unable to provide sufficient spatiotemporal resolution, thereby hindering elucidation of the iterative steps that regulate ECM remodeling during tissue growth and repair over time. The creation of simple and reproducible in vitro environments that replicate multiparametric native 3D cell niches remains challenging.

**Fig. 1.**
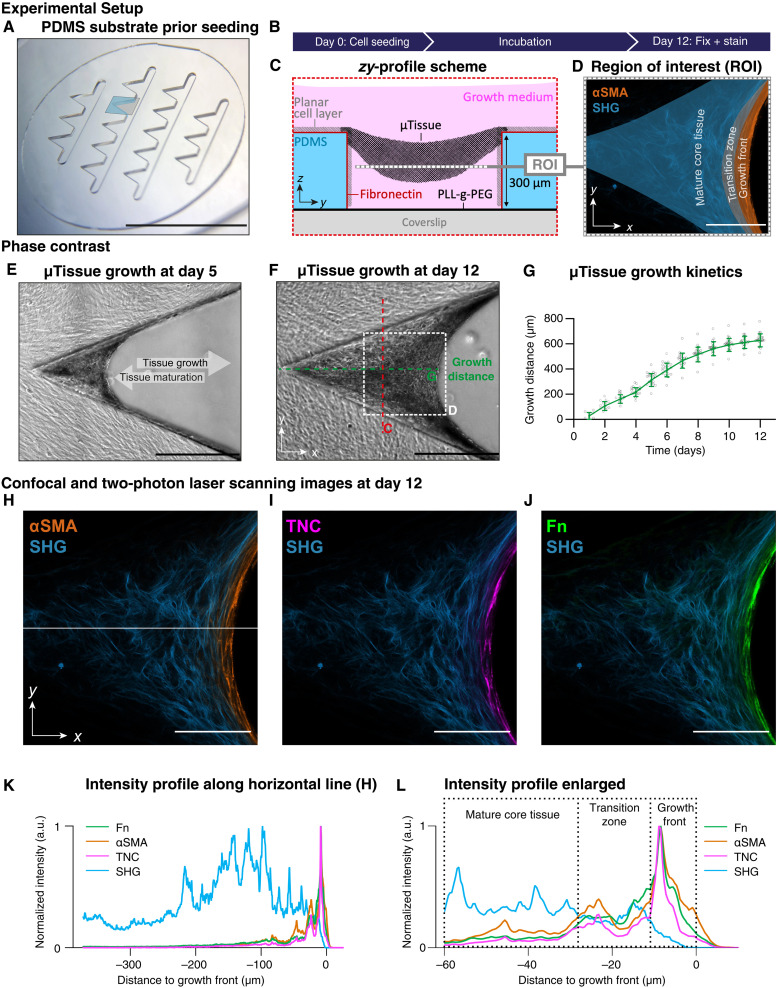
De novo μTissues grown in microfabricated PDMS scaffold arrays. (**A**) Microfabricated PDMS substrate with an array of 16 clefts in which human fibroblasts start to grow 3D μTissues in each cleft (blue color) over time. Scale bar, 1 cm. (**B**) Time course of μTissue growth experiments. (**C**) Profile scheme of the *zy* section [red dashed line in (F)] represents a μTissue freely spanned between the adhesion sites of the functionalized PDMS structures. Region of interest (ROI) indicated by the white dashed line. ROI corresponds to the white dashed line in (D) and (F). (**D**) Confocal and two-photon laser scanning image from ROI in (F) with color overlay indicating growth front (orange), transition zone (gray), and maturing core tissue (blue). Scale bar, 100 μm. (**E**) Phase-contrast image of a cleft with assembled tissue at day 4. The μTissues grow toward the open cleft and tissue maturation occurs over time (gray arrows). Cross-sectioning in the midplane of the μTissues thus provides spatiotemporal information. (**F**) μTissues are fixed and stained at day 12. Scale bar, 500 μm. (**G**) μTissue growth kinetics are plotted as growth distances [dashed green line in (F)] over time. (**H** to **J**) Overlay of αSMA (orange, H), TNC (magenta, I), Fn (green, J), and the SHG signal (blue) after 12 days. Intensity line profiles (K and L) are measured along the white line in (H). Scale bar, 100 μm. More samples in fig. S2. (**K**) Normalized intensity line profile over distance to growth front and along the white line in (H). The absolute gray values are depicted as integrated intensities of the respective profile plots in fig. S6C. (**L**) Enlarged plot of (K) along the first 60-μm distance to the growth front. a.u., arbitrary units.

Here, we used de novo μTissues grown in the corners of millimeter-sized clefts (Fig. 1) as they provide a particularly well-suited 3D model system to investigate how spatiotemporal changes of ECM composition and ECM fiber tension correlate with cell phenotype transitions during tissue growth and maturation (*6*, *43*). Work originating from our laboratory has previously demonstrated that the growth front of such μTissues is rich in myofibroblasts, while the μTissue core is rich in αSMA-negative fibroblasts (*43*). The fibroblasts seeded onto the scaffolds undergo an F-M-T at the growth front even without exogenous TGF-β1 supplementation. Cell proliferation mainly occurs at the myofibroblast-rich growth front, while no indication for apoptotic cells was found (*43*). We therefore proposed that these growth front myofibroblasts later revert back to fibroblasts by undergoing a myofibroblast-to-fibroblast transition (M-F-T), where the cells are increasingly embedded in a collagen fiber network in the tissue core (*43*). Here, we investigated how spatiotemporal changes in ECM composition and architecture, as well as cell contractility and Fn fiber tension, are orchestrated by transiently expressed TNC and TG2 and how all these factors affect cell decision-making. To accomplish this, we advanced the μTissue platform by increasing its throughput, allowing us to perform interventional investigations over a duration of days or weeks. We also developed a semiautomated image visualization tool that allows the comparative evaluation of cell phenotype and ECM marker data obtained with confocal and two-photon laser scanning microscopy. By combining our experimental results with the current literature, we propose a model that describes how the interplay of physical factors and ECM composition might steer the M-F-T. Gaining a better understanding of reciprocal cell-ECM interactions is of major significance to biologists, bioengineers, and the medical community.

## RESULTS

De novo μTissues were grown by seeding normal human dermal fibroblasts onto polydimethylsiloxane (PDMS) scaffolds containing an array of 16 millimeter–sized clefts, each with a cleft angle of 45° (Fig. 1A and fig. S1). Once a confluent 2D cell layer was formed on top of the scaffold after approximately 2 days, the 3D μTissues started to grow in the corners of the clefts, gradually filling the cleft as recorded over 12 days (Fig. 1, B to F). These μTissues were freely suspended in the scaffold cleft (Fig. 1C) as the chamber bottom had been rendered nonadhesive. As the μTissues grew toward the cleft opening, the tissues gradually matured toward the inner corner of the cleft (Fig. 1, D to F). The growth front advanced approximately 60 μm/day during the first 8 days (Fig. 1G).

### μTissue maturation is accompanied by the M-F-T

In agreement with previous reports (*43*), the growth front is rich in αSMA-expressing cells, indicative of a myofibroblastic phenotype (Fig. 1H), while few myofibroblasts are present in the maturing tissue core. Cell proliferation thereby occurs mostly in the growth front, i.e., in the myofibroblast-rich layer that surrounds the tissue, as shown previously (*43*). The proliferative growth front has a width of 10 to 20 μm (Fig. 1K; also compare with different samples in fig. S2) before the newly assembled tissue starts undergoing the M-F-T (Fig. 1H). The rapid change in ECM composition, from the fibrillar Fn–rich growth front to the collagen fiber–rich tissue interior, spatially correlates with the decline of αSMA-expressing cells (Fig. 1, H and J to L, and fig. S2). The Fn fiber density starts to drop steeply within the first 10 μm from the outer μTissue surface, while the collagen fiber density, as visualized by second harmonic generation (SHG), increases rapidly (Fig. 1, K and L). The absolute width of the growth front varies between tissues and is sensitive to various drug treatments, as discussed later (fig. S2). Both the Fn fibers and the myofibroblasts aligned tangentially to the growth front (Fig. 1, H and J). In addition, TNC and TG2 are highly up-regulated in the growth front (Fig. 1I and fig. S3A). The tissue interior consists primarily of collagen-rich ECM (Fig. 1H), sparsely interdispersed with Fn fibers (Fig. 1J), which is in agreement with previous observations of mostly randomly oriented and more rounded fibroblasts in the μTissue core (*43*). Together, the M-F-T coincides in space and time with the appearance of a steep Fn gradient, which colocalizes with rapidly declining αSMA, TNC, and TG2 signals (Fig. 1L and fig. S2).

**Fig. 2.**
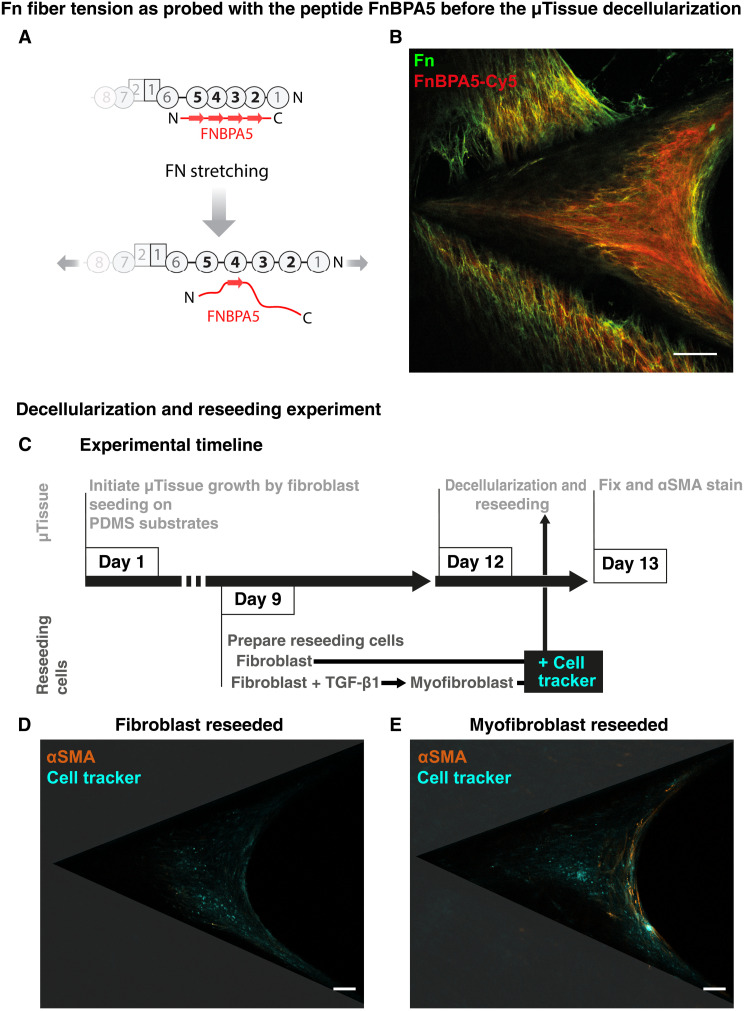
Fn fiber tension and its correlation with the spatial distribution of fibroblasts and myofibroblasts after reseeding decellularized μTissues. (**A**) A large fraction of the Fn fibers are not stretched any longer as probed by the FnBPA5 peptide that binds to structurally relaxed Fn fibers. Relaxed Fn shows higher binding affinity of FnBPA5 due to the availability of multivalent binding epitopes located on the Fn type I domains, while in stretched Fn, the multivalent binding epitope is disturbed, resulting in reduced FnBPA5 binding affinity. Scheme adapted from (*85*) with permission from the terms of the Creative Commons Attribution 4.0 International License, http://creativecommons.org/licenses/by/4.0. (**B**) Superimposed confocal image of Fn (green) and FnBPA5 (red) after 12 days. Scale bar, 100 μm. (**C**) Timeline of reseeding on decellularized μTissue experiment. μTissues were decellularized and reseeded with normal fibroblasts or with TGF-β1–stimulated fibroblasts, which partially converted into αSMA-expressing myofibroblasts. All cells were incubated with cell tracker before reseeding. μTissues were fixed and stained 1 day after reseeding. (**D** and **E**) Central imaging plane of the μTissues fixed after 1 day and visualized for reseeded native fibroblasts (D) and TGF-β1–stimulated fibroblasts (E) with cell tracker (cyan) and αSMA (orange), overlaid by PDMS cleft indications (gray, transparent). For visualization, the αSMA contrast was linearly adjusted using the same absolute values in (D) and (E). In the samples with reseeded native fibroblasts, no αSMA expression was detected with our settings (D), while αSMA-positive myofibroblasts were predominantly localized in the growth front (E). Scale bar, 100 μm.

Complementing our previous Fn–fluorescence resonance energy transfer data and demonstrating highly stretched Fn fibers in the growth front (*43*), we now use our recently developed bacterial adhesin–derived tension probe (FnBPA5), a fluorescently labeled peptide that binds relaxed Fn fibers with substantially higher affinity than stretched Fn fibers (Fig. 2A) (*44*). The μTissue interior showed much higher binding of FnBPA5 compared to the growth front, indicating structurally relaxed Fn fibers in the tissue interior and stretched and partially unfolded Fn fibers in the growth front (Fig. 2B). With this experiment, we confirm that Fn fibers are highly stretched in the growth front, while their tension is released in the tissue core and that the highly tensed Fn fibers colocalize with the myofibroblasts.

### Reseeded myofibroblasts settle in the Fn-rich growth front of decellularized μTissues

To explore how reciprocal cross-talk between the cells and the rapidly transformed ECM might steer the localization of the respective cell phenotypes, we next decellularized μTissues (*45*) and then reseeded the scaffolds with either native fibroblasts or with TGF-β1–stimulated fibroblasts marked with a cell tracker (Fig. 2C). The TGF-β1–stimulated fibroblasts only partially expressed αSMA, resulting in a mixed population of αSMA-positive and -negative cells (fig. S4). The cells were seeded on top of the decellularized ECM and allowed to migrate into the ECM scaffold. Within 24 hours, the reseeded cells (either fibroblasts only or a mixed population of myofibroblasts and fibroblasts) repopulated the entire decellularized μTissue, as indicated by a homogenous distribution of the cell tracker (Fig. 2, D and E) within the representative central z-slice of the μTissue (see relative z-position of the region of interest in Fig. 1C). Importantly though, no αSMA expression was detected 24 hours after cell seeding with native fibroblasts (Fig. 2D). In contrast, reseeding with the mixed cell population resulted in spatial sorting of the cells: While the αSMA-positive myofibroblasts predominantly had relocalized to the Fn-rich growth front region, the cells that had penetrated deeper into the decellularized ECM and repopulated the collagen-rich μTissue core did not express αSMA after 24 hours (Fig. 2E). This suggests that the myofibroblasts preferentially got trapped in the stretched Fn fiber–rich growth front layer but did not continue to migrate into the collagen-rich tissue core.

### EGFR/HER-2 signaling inhibition slows the completion of the M-F-T

**Fig. 3.**
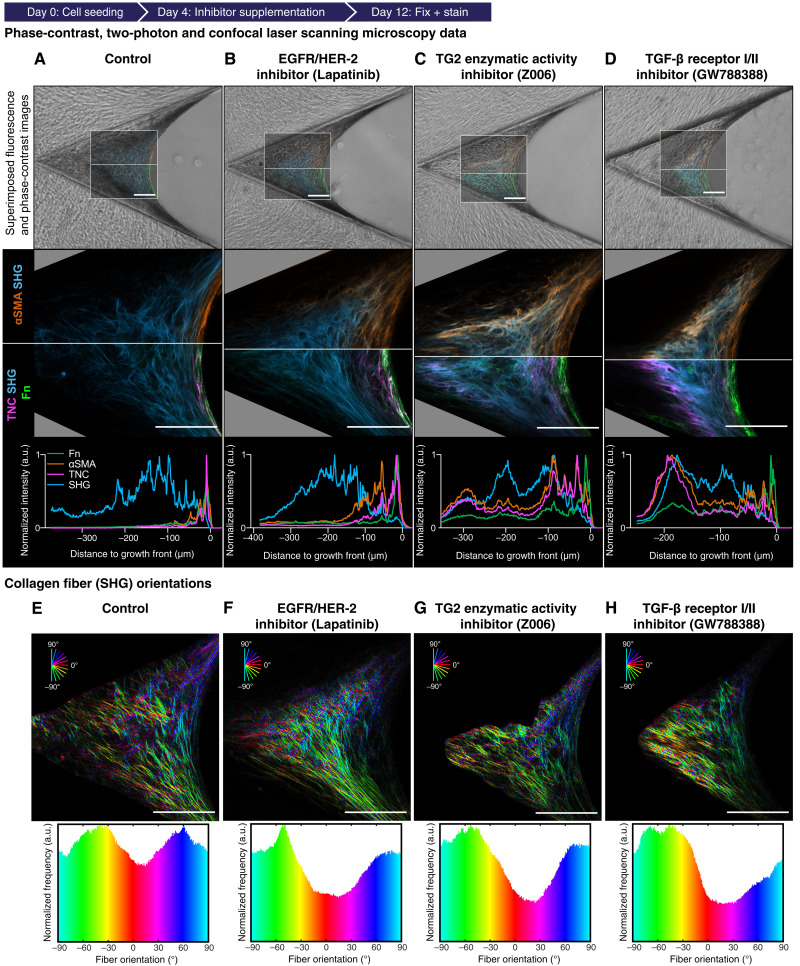
Supplementation of various inhibitors hamper the completion of the M-F-T. (**A** to **D**) Data acquired from 12-day-old μTissues after interventions with various inhibitors from day 4 onward. Top row, representative μTissues imaged with SHG and confocal laser scanning microscope (CLSM) (ROI) and overlaid with the respective phase-contrast image using the following inhibitors: Control [Dimethyl sulfoxide (DMSO), A], inhibition of EGFR and HER-2 signaling (Lapatinib, B), the specific inhibition of the enzymatic TG2 activity (Z006, C), and the inhibition of TGF-β receptor signaling (GW788388, D). Middle row, close-up of the respective ROI in the top row. Since the μTissue grown in the symmetric clefts are expected to be mirror symmetric in their *x* profiles as well, the confocal and two-photon laser scanning microscope (CLSM+SHG) data are depicted with two different sets of look-up table [top LUT: αSMA (orange) and SHG (cyan); bottom LUT: Fn (green), TNC (magenta), and SHG (cyan)]. To visualize tissue morphology, the CLSM+SHG images are contrast-enhanced for each channel to reach saturation in 0.05% of all pixels. Absolute intensities are plotted in fig. S6 (B to F). Scale bars, 100 μm. Bottom row, respective normalized intensity profile of the channel in the middle row is shown parallel to the cleft bisector angle, e.g., main *x*-growth direction of tissue here from left to right (Fig. 1, F and H). At each distance, the mean of 100 vertically accumulated pixels (50 pixels above and 50 pixels underneath the straight line) is plotted over the distance to growth front. See figs. S2, S5, S10, and S11 for more data including separated channels and enlarged profile plots. (**E** to **H**) Top row, collagen fiber (SHG) orientations depicted as pseudo-colored ROI. Bottom row, collagen fiber (SHG) orientations depicted as histogram. See also fig. S7.

What role could the stretching of Fn play and how might it be sensed by the cells? We previously showed that epidermal growth factor receptor (EGFR)/human EGFR-2 (HER-2) signaling is required for mesenchymal stem cells to recognize Fn fiber strain (*17*), whereby stretched Fn fibers up-regulated their osteogenic differentiation. To examine whether alterations in Fn fiber tension contribute to cell decision-making, the previously used dual tyrosine kinase inhibitor of EGFR and HER-2, i.e., lapatinib (GW572016) (*17*), was supplemented to the growth medium of the de novo–grown μTissues. Myofibroblasts are still seen in the growth front of μTissues supplemented with the EGFR/HER-2 signaling inhibitor (Fig. 3B and fig. S5); however, the reversion of myofibroblasts back to fibroblasts (M-F-T) was time-delayed in comparison to the control (Fig. 3A) and was completed only approximately 100 μm away from the growth front. In contrast, tissue growth was not reduced after EGFR inhibition (fig. S6A), suggesting that the overall proliferation rate had not changed. Whether Fn fiber strain regulates either the F-M-T or M-F-T had never been tested before. Our finding that EGFR signaling inhibition substantially delayed the M-F-T suggests that Fn fiber tension sensing by myofibroblasts matters. As this outcome of the EGFR inhibition study would be difficult to explain by potential alterations of morphogenic gradients, these results suggest that the loss of Fn fiber tension in the transition zone affects and perhaps even triggers the M-F-T. This could indicate that not only the recognition of the ECM composition, i.e., Fn versus collagen, but also the ability of myofibroblasts to sense the mechanoregulated alterations in the display of binding sites on Fn fibers, i.e., as displayed by stretched versus relaxed Fn fibers (*14*), might contribute to the stabilization of αSMA expression in the transition zone.

### Inhibitions that hamper the M-F-T also affect the distribution of collagen fiber orientations in the tissue core

Maintaining mechanical tissue stability during tissue growth requires that the contractile forces, as transmitted by cell-cell interactions and via ECM fibers to external anchor points, are counter-balanced by the PDMS scaffold; otherwise, tissue rupture will occur. Cell-generated forces therefore not only regulate intracellular signaling and gene expression programs but also align and stretch ECM fibers. The tissue at the growth front is under substantial tension and collapses upon laser incision (*6*). Images of SHG-positive collagen fiber orientations provide local insights into the tissue force transmission network, as cells arrange the load-bearing collagen fibers along the axes of main force transmission (*46*). A false color mapping of collagen fiber orientations reveals highly aligned collagen fibers behind the growth front but to a lesser extent in the tissue core (Fig. 3E). A large fraction of collagen fibers is also found at angles preferentially parallel to the edges of the PDMS scaffolds (Fig. 3E). In contrast, the collagen fibers in the μTissue core have a more wavy appearance with a random distribution since these collagen fibers intersect each other at many different angles (Fig. 3E and fig. S7A). The highest pixel density of SHG-positive fibers is found in the central region of the μTissues, approximately 100 to 200 μm from the growth front (Fig. 1K). A wavy appearance of collagen fibers, as visualized by SHG, is also a hallmark of healthy tissues (*47*).

A quantitative pixel-by-pixel analysis of collagen fiber orientations further revealed that the fiber orientation histograms of most untreated μTissues are symmetric with respect to the midline of the scaffold cleft (Fig. 3E). However, the fiber orientation histograms were distorted in several samples (fig. S7A), where spontaneous localized tissue ruptures had occurred that loosened the PDMS scaffold anchorage. Such local tissue ruptures release ECM fiber tension, leading to a major rearrangement of collagen fiber orientations in the entire tissue. In addition, the collagen fiber orientation histograms in tissues treated with the EGFR/HER-2 inhibitor in part lose their symmetry, as indicated by the relative lowering of the fiber orientation counts around 0° combined with a prone shift toward one or the other peak (Fig. 3F and fig. S7D). Moreover, the collagen fiber alignment along the growth front is substantially reduced (Fig. 3B and fig. S5). TG2 enzyme activity or TGF-β receptor I/II inhibitions led to similar changes in collagen fiber orientations as compared to the controls (Fig. 3, C and D; figs. S10 and S11; Fig. 3, G and H; and fig. S7, B and C).

### TNC decorates Fn fibers in the growth front but not in the tissue core

What further factors might initiate the abrupt M-F-T accompanied by a concomitant remodeling of the ECM? Immunostaining with a monoclonal antibody revealed that TNC decorates the highly stretched Fn fibers in the growth front (Fig. 1, I and J versus Fig. 2B) but that little TNC was present in tissue core regions (Fig. 1, I, K, and L, and fig. S2). This μTissue finding is in agreement with observations made in cell culture and animal tissues where TNC is rapidly up-regulated in mechanically stressed tissues (*23*, *26*, *34*, *48*) or at injury sites (*36*, *49*) and thus serves as an indicator of active ECM remodeling or immature ECM (*50*). In addition, staining with a polyclonal TNC antibody, which recognizes multiple epitopes, did not identify any TNC or distinct epitopes recognized by the chosen antibodies (e.g., epidermal growth factor-like (EGFL) repeats) in the μTissue core (fig. S8). Our data thus show that TNC is transiently expressed in the growth front and disappears in the transition zone behind the growth front (Fig. 1I and fig. S8), likely by degradation through a variety of different proteases or by autophagy (*36*, *51*).

### TG2 is transiently up-regulated in the growth front, and its inhibition hampers completion of the M-F-T

Like TNC, immunostaining revealed that TG2 was transiently deposited in the growth front, but little TG2 was present in the tissue core (fig. S3A). To ask whether TG2 ECM cross-linking regulates the M-F-T, we inhibited the enzymatic activity of TG2 using the specific catalytic site inhibitor Z006 (*52*). Although Z006 reduced TGF-β1–mediated αSMA expression in 2D cell culture on Fn-coated glass substrates (fig. S9B), αSMA is still expressed in the growth front of Z006-treated 3D μTissues (Fig. 3C and fig. S10). TG2 inhibition with Z006 prevented the elimination of αSMA expression and reduced the down-regulation of TNC in the μTissue cores (Fig. 3, A versus C), resulting in increased absolute fluorescence αSMA and TNC intensities across the tissue (fig. S6B). The immunofluorescence intensity plots revealed that TG2 inhibition leads to a widened but still distinct Fn peak at the growth front (see profile plot in Fig. 3C and fig. S10), as also seen for TNC and αSMA. At the same time, tissue growth was significantly reduced as indicated by the size of the μTissue (fig. S6A). The collagen fiber orientation histograms showed a higher variation in collagen fiber orientations (Fig. 3G and fig. S7B) and less symmetry with respect to the scaffold side walls, suggesting that TG2 inhibition causes more tissue ruptures that resulted in asymmetric force distributions inside the μTissue compared to the control.

We also asked how the opposite situation, the maintenance of artificially high TG2 levels, affects the tissue maturation process. Exogenous TG2 supplementation resulted in reduced μTissue growth (fig. S3, D and E, and fig. S12E), with decreased absolute αSMA, Fn, and TNC intensities (fig. S6H). Relatively high levels of all growth front markers, including αSMA, indicating the presence of a myofibroblast phenotype, persisted in the μTissue core regions (fig. S3D). Thus, suppression of TG2 activity or exogenously supplemented TG2 had unexpectedly similar outcomes, suggesting that the steep gradient of rapidly declining TG2 activity across the transition zone is necessary to complete the M-F-T.

### Inhibition of TGF-β receptor I and II signaling hampers completion of the M-F-T

Since enzymatic ECM cross-linking might locally rigidify the ECM, and as this was suggested to enhance the mechanical release of endogenous TGF-β1 from its latent complex (*53*), we next asked how inhibition of TGF-β receptors I and II might affect the previously described tissue growth and maturation processes. After confirming that TGF-β1 receptor I and II inhibition with GW788388 specifically blocked TGF-β1–mediated αSMA expression in planar 2D cell culture (fig. S9B), GW788388 supplementation did not inhibit αSMA expression in the growth front of the μTissues (Fig. 3D and fig. S11), which was in contrast to expectations derived from 2D cell culture (*11*). Inhibition of TGF-β signaling, however, not only reduced μTissue growth (fig. S6A) but also hampered completion of the M-F-T, as suggested by an enhanced αSMA and TNC expression in the core (Fig. 3D), similar to what was observed with TG2 inhibition (Fig. 3C). Rather than promoting the completion of the M-F-T, TGF-β1 inhibition results in the presence of myofibroblasts in the μTissue core, which is thus remarkable and opposite to what was frequently reported for 2D cell culture (*8*, *11*, *12*). These unexpected differences between 2D cell culture versus 3D μTissues in the cell response to either TG2 or TGF-β receptor I and II inhibitions clearly show that the stabilization of the myofibroblast phenotype in 3D is dominated by other factors than TGF-β1 signaling. Although endogenous TGF-β1 signaling is not crucial for αSMA expression in the growth front of this 3D model system, importantly and unexpectedly, endogenous TGF-β1 signaling, in tandem with other factors, is required to enable completion of the M-F-T.

### ECM remodeling by MMP activity is also required to complete the M-F-T

**Fig. 4.**
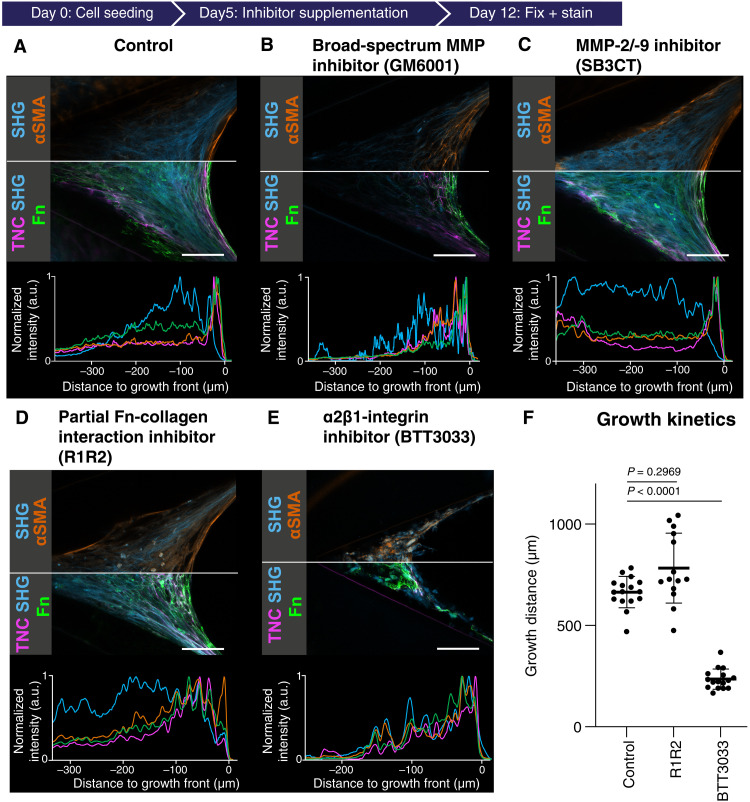
Perturbations with various inhibitors, including inhibiton of MMP activity, Fn-collagen interaction, and α2β1-integrin activity, hamper the proper μTissue maturation. (**A** to **E**) Data acquired from 12-day-old μTissues after interventions with various inhibitors from day 4 onward. Representative μTissues imaged with SHG and CLSM (ROI) under the following conditions: Control (DMSO, A), broad-spectrum MMP inhibition (GM6001, B), specific MMP-2 and -9 inhibition (SB3CT, C), partial inhibition of Fn-collagen interaction (R1R2, D), and a2b1-integrin inhibition (BTT3033, E). Since the μTissues are expected to grow approximately symmetric in their *x* profiles, the confocal and two-photon laser scanning (CLSM+SHG) data are depicted with two different sets of look-up table [top LUT: αSMA (orange) and SHG (blue); bottom LUT: Fn (green), TNC (magenta), and SHG (blue)]. To visualize tissue morphology, the CLSM and SHG images are contrast-enhanced for each channel to reach saturation in 0.05% of all pixels. Scale bars, 100 μm (A to F, bottom rows). Respective normalized intensity profiles of the channels in the middle rows are shown parallel to the cleft bisector angle, e.g., the main *x*-growth direction of tissue here is given from left to right. At each distance, the mean of 100 vertically accumulated pixels (50 pixels above and 50 pixels underneath the straight line) was plotted over the distance to the growth front. See fig. S12 for more samples. (**F**) Growth kinetics visualized as growth distances (in micrometers) at day 12 of control μTissues and of the μTissues treated with R1R2 and BTT3033. Growth distances were measured using the phase-contrast images between the tip of the angular cleft and the μTissue-medium interphase along the bisector angle axis (see Fig. 1F). The significance of differences was tested by performing many-to-one comparisons using Dunn’s test (versus Control).

What could cause the rapid disappearance of TNC and TG2 behind the growth front, coinciding with the M-F-T? TNC was shown to be fragmented by matrix metalloproteinase (MMP) -1, -2, -3, and -7 (*36*). Furthermore, the broad-spectrum MMP inhibitor GM6001 (or Ilomastat), inhibiting MMP-1 to -3, MMP-7 to -9, MMP-12, and MMP-26 was shown to block TNC degradation in vitro (*54*). In GM6001-treated μTissues, while having little impact on the growth front that continued to show αSMA-positive cells, as well as Fn and TNC, the μTissue core was mostly depleted of SHG-positive collagen fiber bundles (Fig. 4B) compared to the control tissues (Fig. 4A and fig. S12A). The absolute intensities of Fn, αSMA, TNC, and the SHG signal were greatly reduced across the entire μTissue profile (fig. S6G). In addition, TG2 deposition also remained up-regulated in the GM6001-treated μTissue cores (fig. S3C and fig. S13C). In contrast to the broad-spectrum MMP inhibition with GM60001 (Fig. 4B), selective MMP-2 and MMP-9 inhibition with SB3CT (*54*) had little impact on μTissue morphology (Fig. 4C and fig. S12B). Collagen fibers formed normally (Fig. 4C), and the total fluorescence intensities were similar to those in the control tissues (fig. S6G). MMP-2 and MMP-9 inhibition with SB3CT did not result in alterations in the Fn-, TNC-, or αSMA-gradient slopes, or in alterations in F-M-T or M-F-T (Fig. 4C, profile plot). Together, broad-spectrum MMP activity, other than MMP-2 and MMP-9 alone, are required for the formation of steep TNC, Fn, and αSMA gradients in the transition zone and to assemble a mature collagen fiber network in the core region. The fact that MMP inhibition shows major effects illustrates that these proteolytic enzymes are locally secreted and active and that their activity is highly regulated in space and time as MMP activity is crucial for tissue remodeling.

### Disruption of Fn-collagen interactions hampers tissue maturation

Since the initiation of collagen assembly requires the presence of Fn fibers as template (*4*, *5*, *55*), we next investigated how the partial inhibition of Fn-collagen interactions affects the onset of the tissue maturation process. We thus partially inhibited Fn-collagen interactions through competitive binding of the supplemented bacterial adhesin–derived R1R2 peptide to the collagen binding site on Fn (*56*, *57*). A pronounced but widened growth front rich in Fn, TNC, and αSMA was still formed (Fig. 4D), and the tissue growth rate was not significantly altered (Fig. 4F). However, TNC and Fn degradation and replacement by a mature collagen–rich matrix were substantially delayed and incomplete, while αSMA remained elevated in the core as well (Fig. 4D and fig. S12D). This could be due to a reduced degradation or enhanced expression of TNC and Fn in the core. Overall, the M-F-T occurred but was incomplete, suggesting that the Fn-collagen interaction is essential for myofibroblast clearance (Fig. 4D). Compared to the control (movie S1), many μTissues showed detachment from the side walls of the clefts and internal ruptures upon R1R2 peptide treatment [movie S2 (R1R2)], indicating a strong reduction of mechanical tissue stability. Most of the tissues demonstrated similar cleft closing properties compared to control tissues. Together, this suggests that collagen binding to Fn fibers in the transition zone is required to assemble SHG-positive collagen fibers in the core and thus to reinforce the mechanical stability of the μTissues. The presence of SHG-positive collagen fibers in the core is also required for the myofibroblast clearance to occur in the core.

### Inhibition of α2β1 integrin–mediated cell-collagen binding hampers tissue growth and maturation

The presence of the major cell-collagen–binding integrin α2β1 is essential to initiate collagen fiber assembly and, subsequently, the formation of dense collagen fiber networks (*4*, *58*). To investigate the effect of α2β1integrin blocking on collagen fiber network assembly, we next treated 4-day-old μTissues with BTT3033, an α2β1-integrin inhibitor. Although αSMA was expressed, no clear αSMA-positive growth front was identifiable (Fig. 4E and fig. S12C), and tissue growth was significantly reduced upon exposure to BTT3033 (Fig. 4F) compared to the control (Fig. 4A). An SHG signal was visible, but the formation of organized SHG-positive fibers was mostly suppressed, with the SHG signal appearing as amorphous “blebs” rather than organized fiber structures (Fig. 4E). All growth front markers were distributed throughout the entire tissue, and a spatial transition from growth front to mature core region was lost. Together, α2β1 integrin–mediated cell binding to collagen is crucial to initiate the ECM remodeling, from a Fn-rich growth front to a collagen-rich core, and thus for a sharp M-F-T to occur.

### The sequence of events observed in regions of repair following spontaneous μTissue rupture resembles the growth front

**Fig. 5.**
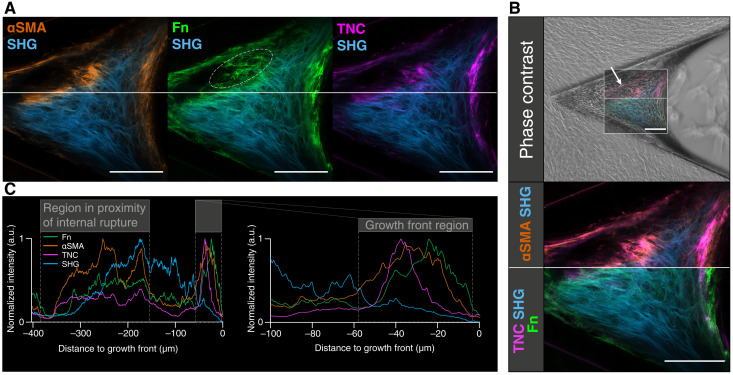
Tissue repair after spontaneous internal tissue ruptures of μTissues. (**A**) Representative ROI of μTissues that had spontaneously ruptured as imaged with confocal and two-photon laser scanning microscope (CLSM+SHG) after 12 days. The CLSM+SHG data are stained for αSMA (orange), Fn (green), and TNC (magenta); SHG visualized in blue. The early onset of repair can be seen in the Fn channel, where a new Fn matrix was assembled within the ruptured void (white dashed line). Scale bars, 100 μm. (**B**) Representative μTissue with spontaneous rupture imaged with SHG and CLSM (ROI): overlaid with the correlating phase-contrast image (top row), and a close-up of the ROI (bottom row). The CLSM+SHG data are depicted with two different sets of look-up table [top LUT: αSMA (orange) and SHG (blue); bottom LUT: Fn (green), TNC (magenta), and SHG (blue)]. Scale bars, 100 μm. (**C**) Intensity profile plots along the horizontal line as the column average of 100 pixels indicate the similarity between the growth front markers and the pattern at regions in proximity to the internal rupture.

During tissue growth, some μTissues showed spontaneous internal ruptures with loss of internal tissue continuity in regions facing the cleft edges (Fig. 5, A and B). The repair processes initiated in the surrounding tissues showed an accumulation of the growth front markers αSMA, TNC, and Fn, similar to that observed in the actual growth front (Fig. 5C). This suggests that normal growth and maturation processes are recapitulated during tissue repair. Rapid reestablishment of mechanical stability is essential after tissue rupture or injury. Here, the same spatiotemporal appearance of myofibroblasts and expression of key molecular players that orchestrate the M-F-T were observed during tissue repair and tissue growth. This also illustrates that this μTissue platform is well suited to investigate spontaneous tissue ruptures and subsequent repair processes over time.

## DISCUSSION

**Fig. 6.**
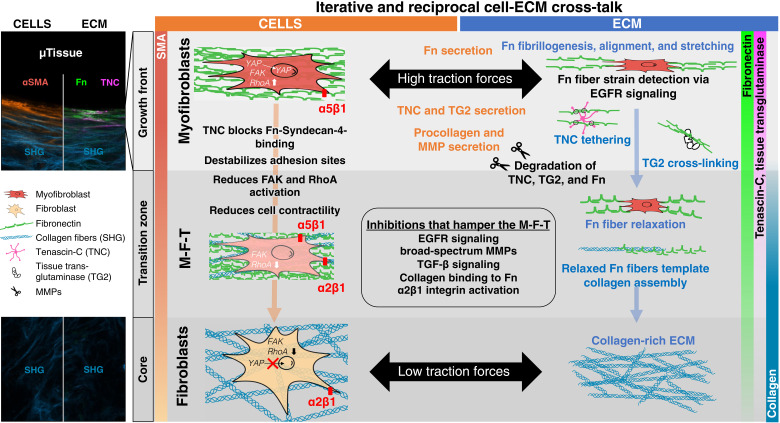
Overview figure of proposed events that orchestrate μTissue growth and maturation. Together with the existing literature, our data suggest that the M-F-T is regulated by a sequence of iterative and reciprocal interactions between cells (left) and the ECM (right). Through up-regulated FAK/RhoA activation, α5β1-integrin signaling leads to a build-up of a more contractile actomyosin cytoskeleton, which promotes the translocation of the nuclear transcription factors YAP/TAZ from the cytoplasm to the nucleus (*86*), stimulating profibrotic gene expression, including TNC (*63*). As a result, highly proliferative myofibroblasts exert high contractile forces and assemble the first Fn matrix and stretch the Fn fibers. The highly tensed microenvironment at the growth front stimulates myofibroblasts to secrete a variety of proteins, including TNC and TG2, which physically (TNC) and enzymatically (TG2) cross-link and stabilize the early Fn ECM. TNC inhibits cellular contractility and its own expression by blocking syndecan-4 binding to Fn and down-regulating downstream FAK and RhoA/ROCK signaling (*72*). At the same time, Fn fiber strain decreases in the transition zone, increasing Fn-collagen binding and optimizing the role of Fn as template for collagen fiber and network assembly (*5*), as the tissue matures and M-F-T occurs. Last, a switch from the Fn integrin α5β1 to the collagen integrin α2β1 is necessary for the cells to interact with their changing ECM environment. These time-staged iterative and reciprocal interactions between cells and their ECM are crucial to enable proper tissue growth and maturation. All pharmacological interventions performed either delayed or abolished the normal tissue maturation and M-F-T processes described here (see Figs. 3 and 4 and figs, S2, S3, S5, and S10 to S13).

By using de novo–grown μTissues in engineered PDMS cleft arrays (Fig. 1, A to D), we identified crucial actors and events that modulate reciprocal cell-ECM interactions and thereby iteratively regulate tissue growth and cellular phenotype control. The observations reported here are likely not restricted to dermal fibroblasts, as such μTissues were previously also grown from preosteoblastic cells and showed similarly abrupt changes from a Fn fiber–rich front to a collagen-rich core (*6*, *59*). It was shown that human dermal fibroblasts transition into highly contractile and proliferative myofibroblasts at the μTissue growth front, while cell divisions are rare in the fibroblast-rich core (*43*). A rapid transition toward a fibroblast-rich maturation zone then takes place, which thus requires a transition of myofibroblasts into fibroblasts (M-F-T) (*43*). To shed light onto underpinning processes, we analyzed how iterative reciprocal cross-talk between cells and their newly formed ECM regulates the M-F-T (Fig. 1H) and found that the transition coincides with a rapid remodeling of the ECM (Fig. 1, H to L). Since a direct analysis of local and steep gradients of morphogens and other soluble factors is difficult, particularly across the transition zone, we used various pharmaceutical inhibitors. These revealed that the spatial sharpness of the transition zone requires the transient up-regulation of both the matricellular protein TNC and the cross-linking enzyme TG2 in the growth front, followed by their rapid clearance with high spatiotemporal precision (Fig. 3A), but also EGFR signaling (Fig. 3B), MMP activity (Fig. 4B), collagen templating on Fn (Fig. 4D), and α2β1 integrin activation (Fig. 4E). Inhibiting any of these processes prevents the tissues from completing the M-F-T, as suggested by increased αSMA levels in the tissue core. Taking all of our observations together with the literature, we propose that the M-F-T requires the following actors and iterative steps by which the transition from tissue growth to maturation is sequentially controlled (Fig. 6).

### Growth front

The highly tensed growth front is only a few cell layers thick, and the first provisional matrix freshly assembled there by myofibroblasts consists of highly stretched Fn fibers aligned parallel to the growth front (Fig. 1J), as well as of transiently up-regulated TNC (Fig. 1I) and TG2 (fig. S3A). Some myofibroblasts are highly mobile and migrate on the μTissue surface (movie S1) while assembling Fn fibers, similar to cell behavior observed at sites of tissue repair after punching holes in cell-seeded collagen gels (*60*). The cells in the growth front secrete cellular Fn that also contains the ED-A domain, among others, known to increase αSMA expression, which, in turn, up-regulates cell contractility and enhances ECM deposition (*61*). On the basis of the spreading of the proliferating myofibroblasts at the outer edge, the μTissue advances and we propose that the highly stretched and aligned ECM fibers at the growth front stabilize the myofibroblast phenotype, in agreement with the literature (*62*). Spatial distribution of αSMA-positive cells reseeded onto decellularized μTissues suggests that myofibroblasts, in contrast to fibroblasts, potentially migrate with preference into the space characterized by aligned and highly stretched Fn fibers but not further into the tissue (Figs. 2, B and E, and 3B). A difference in migration speed between the two cell phenotypes may have also helped their segregation in the microenvironment. The preferential localization of fibroblasts and myofibroblasts at the middle position of the μTissue (white dashed line in Fig. 1C indicates the middle imaging plane) supports that the ECM characteristics in the growth front have a considerable contribution to the spatial sorting of these cell types. This indicates that both cell types are capable of migrating from the top surface into the μTissue as they are found in the middle imaging plane in different regions. However, the spatiotemporal dynamics of the reseeded cells in such a spatially heterogeneous microenvironment remains to be elucidated in future investigations to fully decipher the individual contributions of cell type–dependent migration and external ECM characteristics. High traction forces applied by the myofibroblasts to their environment, or vice versa, are known to up-regulate the expression of TNC (*23*), which is a YAP target gene (*63*) and explains the appearance of TNC in the growth front (Fig. 1I). (Myo)fibroblast-mediated contractile forces were proposed to activate TGF-β1 by mechanically releasing it from its ECM-bound latent binding protein (LTBP-1) (*11*). This mechanical TGF-β1 release is especially effective when the LTBP-1 is bound to an ECM under tension that counters cell-applied forces, such as in the μTissue growth front (*64*). TGF-β1 expression up-regulates both TG2 (*65*) and TNC (*34*) expression. Endogenous TGF-β1 release in the growth front region might therefore elevate local TG2 and TNC expression (Fig. 1I and fig. S3A).

The Fn-rich provisional ECM of growing tissues, or at the edges of wounds, is often exposed to major mechanical agitation that can easily disrupt the newly emerging provisional ECM. This raises the question how such a provisional Fn matrix can be rapidly mechanically reinforced. TNC might play a crucial role in the initial mechanical growth front stabilization since TNC is secreted at high concentrations in the growth front (Fig. 1, I and L) and has a star-like conformation, with each of its six arms containing a Fn binding site that can bind to three different regions of Fn (Hep I, Hep II, and Hep III domains) (*23*). We propose that TNC can tether Fn fibers that are assembled and bundled parallel to the growth front (fig. S2) and are thus in proximity to each other, i.e., within distance of physical reach of individual TNC arms. Here, the recently identified MAREMO interaction sequence in TNC and FN could be relevant (*66*). Whether TNC in the μTissue growth front is still hexameric is unknown, nor has a direct in vivo tethering function of Fn fibrils by TNC been suggested. However, multiple interactions of FN with TNC were recently described (*66*). Such physical tethering could mechanically stabilize the early Fn fiber network in the growth front. TNC might also serve as a shock-absorbing element since the FnIII domains in TNC display similar mechanical stability to those in Fn and allow TNC to be stretched to multiple times its resting length (*24*, *25*). In addition, TNC is a TLR4 activator that up-regulates the expression of various profibrotic effectors, including TGF-β, αSMA, Fn, and Coll-1 but also TNC itself (*16*). As a result, TNC and TLR4 can drive each other’s expression and activation (*37*), which, together with the spatial distribution of TNC in the μTissues (Fig. 1K), emphasizes the highly complex and reciprocal events starting in the growth front region. Fn and TNC can also bind soluble factors, such as TGF-β1 itself, and thus generate a reservoir for these molecules (*18*, *66*–*68*).

TG2 is also up-regulated in the first cell layers in the μTissue growth front (fig. S3). In contrast to the noncovalent physical tethering by TNC, TG2 can chemically cross-link Fn and other ECM fibers through formation of intermolecular or intramolecular isopeptide bonds. Myofibroblast-generated uniaxial forces parallel to the growth front drive the bundling of Fn profilaments (*69*), thereby providing the close physical contact between fibers required for isopeptide bond formation by TG2. We therefore propose that TG2 chemically cross-links and stabilizes the early Fn ECM in the growth front in de novo–grown μTissues. As the tethering and cross-linking of Fn fibers proceed, the cross-linked Fn fiber bundles are slowly buried underneath new layers of proliferating myofibroblasts, thus approaching the transition zone.

### Transition zone

Fn fibers lose their tension (Fig. 2B) and both TNC and TG2 concentrations decline (Fig. 3A and fig. S3A) in a less than 20-μm-wide, sharp transition zone. All growth front markers (αSMA, Fn, TNC, and TG2) decline rapidly as the distance to the growth front increases (Fig. 1, H to L, and figs. S2, S3A, and S13A). This indicates a tightly synchronized switch of both the ECM composition and the cell phenotype. Once the newly expressed proteins, including TNC and TG2, are incorporated into the ECM, the cells respond in a first feedback loop and remodel the ECM, perhaps aided by the transient expression of specific soluble factors and by binding of these factors to FN, TNC, and other ECM molecules (*66*, *67*). However, what regulatory mechanisms could be involved in the initiation of the rapid conversion of myofibroblasts to fibroblasts, and how could the nonstructural proteins that are transiently up-regulated in the growth front contribute to this conversion? The appearance of TNC in the growth front is expected to suppress RhoA and focal adhesion kinase (FAK) activity and thus decreases contractility by competing with syndecan-4 for Fn binding (*70*, *71*). This feature might shield the early ECM from damage through excessive myofibroblast-driven cell contraction and facilitate movement of activated fibroblasts within the early wound matrix. In a self-limiting feedback loop, the presence of TNC in the ECM inhibits its own expression by blocking syndecan-4 binding to Fn and down-regulating downstream RhoA/Rho-associated protein kinase (ROCK) signaling and subsequent TNC expression (*27*, *70*, *72*). Thus, in addition to TNC degradation, a reduction of TNC expression could explain the TNC decline in the transition zone (Fig. 1I). Importantly though and unexpected from most of the 2D cell culture literature (*8*, *11*, *12*), exogenous TGF-β1 supplementation resulted in increased μTissue volume (*43*) but did not affect the overall phenomenon of M-F-T and growth front marker disappearance toward the tissue core and, thus, the sequence of tissue growth and maturation events (*43*). This unexpected observation agrees, however, with cell reseeding experiments into 3D ECM made by cancer-associated fibroblasts showing that ECM fiber alignment stabilizes the myofibroblast phenotype independent of TGF-β1 supplementation (*62*). Here, our pharmaceutical interventions through TGF-β1 receptor I and II inhibition with GW788388 and TG2 enzymatic activity inhibition with the specific catalytic site inhibitor Z006 both showed hampered completion of the M-F-T since growth front markers, including αSMA, were still present in the tissue interior (Fig. 3D). Exogenous supplementation of TG2 also inhibited the M-F-T and associated ECM remodeling as high levels of αSMA, Fn, and TNC persisted in the tissue core (fig. S3D).

It was unexpected that both the inhibition of enzymatic TG2 activity and the supplementation of exogenous TG2 have similar outcomes, which points to the importance of a highly localized endogenous TG2 expression followed by a subsequent timely depletion. This furthers the notion that TG2 displays its ECM cross-linking activity at a specific time and place and that TG2’s cross-linking activity needs to be turned off soon thereafter to allow rapid ECM remodeling in the transition zone. When enzymatic TG2 activity is blocked, which should mechanically destabilize the ECM, we find that TNC is present throughout the tissue core. To maintain mechanical stability, we propose that the system might partially compensate for the loss of TG2’s cross-linking activity in the growth front by up-regulating physical ECM tethering by TNC in the tissue core (Fig. 3C). This suggests a compensatory prevention of the degradation or down-regulation of TNC in the transition zone as a result of blocking TG2’s enzymatic activity. As such, both TNC and TG2 seem crucial for the myofibroblast-to-fibroblast conversion.

As the Fn, TNC, and TG2 levels decline steeply in the transition zone, what might be the role of endogenously expressed MMPs in regulating the M-F-T and the rapid remodeling of the ECM in the transition zone? Usage of a broad-spectrum MMP inhibitor prevented the degradation of TNC (Fig. 4B and fig. S12A) and of TG2 (fig. S3C and fig. S13C), in agreement with the literature (*36*, *54*, *73*), and resulted in reduced total collagen deposition (fig. S6G). In contrast to broad-spectrum MMP inhibition, selective MMP-2 and MMP-9 inhibition with SB3CT (*74*) had little effect (Fig. 4C and fig. S12B). While MMP-2/-9 activity seems not to be crucial for the relevant tissue remodeling processes, the overall activity of other MMPs is crucial for the rapid remodeling of the early Fn matrix into the maturing core tissue and thus paves the way for the M-F-T to occur.

MMP-1, -2, -3, -7, and -9 are known to cause the fragmentation of TNC (*36*) and the degradation, turnover, and endocytosis of Fn (*75*, *76*). These data suggest that high levels of endogenously produced MMPs in the growth front trigger the subsequent fragmentation of TNC and Fn in the transition zone. TNC fragments could thereby convey opposite functionality: Full-length TNC promotes migration via its antiadhesive function, but TNC fragments were shown to inhibit cellular migration (*3*, *77*). This could explain the transition of highly migratory cells in the outer growth front toward more stationary cells in the core, which would imply that a protease-dependent switch in TNC functionality is crucial for completing the M-F-T. In addition, Fn fragments have other biological activities than the full-length molecules (*78*). The degradation of stretched Fn fibers in the growth front might be reduced under MMP inhibition (*75*, *76*). As MMP-mediated mechanical unloading of Fn fibers is expected to increase the number of relaxed Fn fibers available as template for collagen assembly (*5*), this could explain the substantial reduction of SHG-positive collagen fibers under broad-spectrum MMP inhibition (Fig. 4B and fig. S12A). This would imply that a protease-dependent switch in Fn fiber tension is crucial for tissue maturation. Together, our data suggest that broad-spectrum MMP activity facilitates the partial degradation of the Fn-rich provisional matrix in the transition zone and paves the way for the subsequent ECM remodeling toward a collagen-dominated maturing matrix.

What additional tasks could be regulated by alterations of Fn fiber tension (*14*) beyond templating collagen assembly (*5*)? Does the tensional state of the Fn fibers matter in stabilizing the myofibroblasts in the growth front and in regulating tissue maturation or the completion of the M-F-T? Previous data suggest that osteogenesis is up-regulated for mesenchymal stem cells on stretched Fn fibers and that their ability to sense Fn fiber strain is lost upon EGFR/HER-2 inhibition (*17*). In de novo–grown μTissues, the inhibition of EGFR/HER-2 signaling had little effect on the width of the Fn-rich growth front regions but greatly delayed the disappearance of the αSMA-expressing myofibroblasts (Fig. 3B and fig. S5), without a notable change in growth rate (fig. S6A, lapatinib). Together, this indicates that the inability of myofibroblasts to probe Fn fiber tension delayed the M-F-T yet had little effect on their proliferation rate. The observed loss of Fn fiber stretch sensitivity could be due to the loss of TLR4 signaling, as the TLR4 binding site on the ED-A domain of cellular Fn is cryptic and might get exposed by Fn fiber stretching (*14*, *15*). We also know that the supplementation of the Fn ED-A fragment to 2D cell culture drives collagen gene expression and myofibroblast transition through TLR4 signaling (*16*, *79*) and that TNC supplementation to TLR4-deficient fibroblasts showed reduced αSMA and collagen expression (*37*). Alternatively, one could argue that EGFR autophosphorylation can also be induced by binding to the EGF-like repeats of TNC (*80*) and suggest that the appearance of TNC in the ECM might stimulate EGFR signaling and thus augments Fn fiber strain sensing. While the latter argument regarding the role of TNC might explain the Fn fiber tension sensing in the growth front, this does not explain why the onset of the M-F-T is so much delayed upon EGFR signaling inhibition, as αSMA expression is still seen 100 μm away from the growth front even beyond the point where TNC has already mostly disappeared (Fig. 3B). Sensing of the Fn fiber strain by the myofibroblasts, perhaps involving other mechanoregulated binding sites as well, is thus crucial for the timely onset of the M-F-T to occur.

### Completion of the M-F-T and onset of tissue maturation

Upon the appearance of collagen fibers, the cells respond in a next feedback loop (Figs. 1H and 6). As more ECM proteins appear, outside-in cell signaling is switched from first engaging the Fn integrin α5β1, then also the binding to TNC via integrins αvβ3 and α9β1, and finally interacting with collagen I via integrin α2β1. We observed that reduced αSMA expression in the transition zone coincides with the appearance of structurally relaxed Fn fibers (Fig. 2B) and increasing amounts of SHG-positive collagen fibers (Fig. 1J). This is expected as only structurally relaxed but not stretched Fn fibers can serve as a template for the nucleation of collagen fiber assembly (*5*, *55*). Upon tissue maturation, the tissue core is mechanically stabilized by bundles of densely intersecting and randomly oriented collagen fibers (Fig. 4, A and E). Those collagen fibers show a much more wavy appearance, while the collagen fibers at the μTissue peripheries are aligned mostly parallel to the tissue edges (Fig. 4A and fig. S2). The appearance of randomly oriented and wavy collagen fibers is a signature of healthy tissues (*47*), while the persistence of highly aligned collagen fiber bundles is typically found in situations of major ECM remodeling like in fibrosis and cancer (*62*). Furthermore, partial blockage of Fn-collagen interactions via the peptide R1R2 in 4-day-old μTissues (*5*, *56*, *57*) indeed did not prevent further tissue growth (Fig. 4F) but destabilized the collagen fiber architecture in the μTissue core, which resulted in much more frequent tissue rupture events, even causing partial tissue detachments (movie S2). In addition, αSMA and TNC remained elevated in the core (Fig. 4E and fig. S12D), perhaps indicating an up-regulation of localized tissue repair responses. Since fibroblasts bind to collagen via integrin α2β1, 4-day-old μTissues were treated with the α2β1-integrin inhibitor BTT3033. Upon BTT3033 treatment, tissue growth was significantly reduced (Fig. 4, E and F), with high tissue size variability between samples (fig. S12C). All growth front markers were distributed throughout the entire tissue, and a sharp spatial transition from growth front to mature core region was lost. Such a shift in engaged integrins is expected to reduce cell contractility as Fn depletion down-regulates FAK and RhoA activity and, thus, of cell contractility in the core, thereby reducing the energy demand and metabolic activity of the resident fibroblasts (*81*). Last, we would like to point out that the core of our μTissues is filled with structurally relaxed Fn fibers (Fig. 2B). In contrast, most healthy organ tissues that we have investigated so far contain highly stretched Fn fibers, while major Fn fiber relaxation is seen in cancer (*82*). Nevertheless, our model system is well suited to explore factors that regulate the M-F-T in the absence of paracrine signals secreted by other cell types, including immune cells and blood vessels.

Together, how could this iterative process as regulated by reciprocal feedback loops work? We would like to propose the following: While myofibroblasts create a unique tissue repair microenvironment in the growth front, where they assemble and stretch Fn fibrils, they soon respond at the transcription level to the self-generated tension (Fig. 6). Because of their high contractility, they enter the first round of feedback, which requires time to be initiated as up-regulating the expression of proteins is not instantaneous. This also includes the up-regulated expression and secretion of structural and nonstructural proteins, including TNC, TG2, TGF-β, and MMPs. Their local appearance then launches the next cycle of reciprocal feedback that pushes the system toward the transition zone. These transiently expressed proteins act with high spatial and temporal precision and need to be degraded to ensure the completion of the M-F-T, thus allowing a collagen-rich core to mature without being perturbed by the presence of αSMA-expressing cells. In this first round of feedback changes in gene expression, secretion of these proteins is thus delayed, and the consequences thereof dominate the time-dependent events seen in the transition zone. Toward initiating the onset of the transition zone, our data suggest that several processes contribute synchronously, and interference with any one of them hampers the timely completion of the M-F-T: Myofibroblast sensing of the Fn fiber tension is as important as the timely ECM remodeling, which requires the degradation of TNC and TG2. The loss of Fn fiber tension in these μTissues can have various reasons, including MMP-mediated Fn fragmentation or reduced cell adhesiveness to Fn fibers due to TNC. Reduced interconnectivity within the ECM fiber network resulted in tissue rupture events. The sequence of events described here is not altered by the inhibition of TGF-β receptors (Fig. 3D and fig. S11) or by its exogeneous supplementation (*43*), but only tunes their kinetics. While this is in contrast to observations made in 2D cell culture, it agrees with observations made when reseeding fibroblasts into decellularized cancer-associated fibroblast-derived ECM, where TGF-β supplementation was not necessary to induce αSMA expression (*62*). Therefore, factors that reduce the mechanical stability of the ECM fiber network by any of the interventions tested here are expected to lead to localized tissue ruptures as cells pull on the ECM fibers. Within the ruptured tissue regions, the growth front markers αSMA, TNC, and Fn appeared in the immunostains (Fig. 5), demonstrating that the observed tissue growth and maturation processes are recapitulated during tissue repair.

In conclusion, we have identified how various actors, which regulate tissue growth and maturation processes, play together under high spatial and temporal control, fine-tuned by multifaceted reciprocal dependencies (Fig. 6). The actions of tissue fiber tension, together with transiently up-regulated matrisomal proteins, including TNC and TG2, as well as of TGF-β and proteolytic enzymes, such as MMPs, demonstrate a precisely timed functional complementarity and together stage the onset and completion of the M-F-T. Together with the literature, our data further suggest a high relevance of Fn fiber tension in modulating these processes. The sum of our experimental observations emulates into a conclusive picture. All tested pharmacological inhibitors that interfere with one of the central steps of tissue maturation led to delayed or incomplete conversion of myofibroblasts to fibroblasts (Fig. 6). This phenotype reversion is of utmost importance as failed phenotype reversion and prolonged myofibroblast activity at wound sites leads to dysfunctional fibrotic tissues (*10*, *11*). The timing of the events is complex. Some cellular actions that are downstream of integrin engagement in response to ECM alterations are instantaneous, while others require changes in gene expression and are thus time-delayed. Iterative reciprocal feedback loops between the cells and their self-made ECM as illustrated in Fig. 6, each requiring characteristic time windows for their implementation, are thus central to the sequential maturation of the growth front tissue and enable the sharpness of the transition zone.

Our findings might have far-reaching implications, as the persistent occurrence of myofibroblasts located in proximity to aligned SHG fibers are hallmarks for fibrotic pathologies and are found in the vicinity of tumor cells, where they are considered to be key drivers of tumorigenesis and cancer progression via modulation of the ECM (*12*, *30*, *62*, *82*). Our in vitro–grown model tissues allow the tuning of selected parameters that cannot be easily tuned in vivo. Future studies using more complex cell co-cultures can explore how the sequence of events described here might depend on the presence and cross-talk with other cell types. A better understanding of the mechanisms driving phenotype reversal is not only of fundamental interest in the biosciences but also crucial for the development of novel therapeutic strategies. In the future, our platform, combined with fibroblasts harvested from patients, may help to study personalized factors influencing ECM gradients, tissue growth, and maturation. Such knowledge can contribute to the development of novel diagnostic and personalized treatment strategies with relevance to fibrotic and neoplastic diseases. In summary, tissue growth is delicately organized in time and space, like a well-orchestrated symphony, where different actors are transiently required, and their individual actions are switched on and off in time.

## MATERIALS AND METHODS

### Experimental design

Our studies were performed using de novo 3D μTissue grown in millimeter-sized clefts of PDMS substrates, and we investigated specific pharmaceutical interventions by supplementing inhibitors to the growth media. μTissues were incubated for 12 days, and chemical inhibitors were supplemented to the PDMS substrates at day 4 of tissue growth.

### Rationale for substrate design and fabrication method

PDMS substrates were fabricated by replica molding of negative SU8-master structures, improving the previously published protocol (*43*). To avoid residual membrane formation, a custom-built compression device was applied as described below. To reduce μTissue-to-μTissue variability, the cleft edges were smoothed with a photomask of higher resolution, and each cleft is separated so that the grown μTissues are not in direct contact with each other. Each substrate consists of a cluster of 16 clefts arranged in arrays. The arrangement of multiple cluster replicas on one master mold allowed a higher production throughput. Since it was shown that geometry can coordinate tissue growth (*59*), the cleft angle of 45° was chosen to reduce incubation times and still achieve sufficient tissue growth.

### Master fabrication

A master mold was fabricated by standard lithography of multiple layers of SU8-3050 photoresist (MicroChem, USA). In brief, a 4″ silicon wafer was dehydrated at 120°C for 10 min. For the first layer, 2 ml of SU8-3050 was dispensed and spin-coated at 500 rpm for 10 s with an acceleration of 100 rpm/s followed by 1000 rpm for 45 s with an acceleration of 500 rpm/s. After spin coating, a soft bake at 65°C for 7 min and at 95°C for 15 min was performed. After cooling down to room temperature (RT), spin coating was repeated twice with 4 ml of photoresist, each resulting in a ~350-μm-thick resist layer. Samples were stored at RT for 24 hours before exposure. For exposure, a photolithography mask with a resolution of 32,000 dpi (Zitzmann GmbH, Germany) and an MA6 mask aligner (Karl Suss, Germany) were used, and the samples were exposed with a total dose of 400 mJ/cm^2^ (λ = 365 nm) divided into four runs with a 60-s pause in between exposures. Post-exposure bake was performed at 65°C for 3 min and at 95°C for 10 min. Because of the thickness of the resist, all heating and cooling steps were performed at slow ramp. The resist was developed using mr-Dev 600 developer (Microresist Technologies, Germany) under ultrasound for 20 min, followed by a wash with fresh developer and isopropanol, each for 10 s. Master molds were passivated with 20 μl of trichloro-(1*H*,1*H*,2*H*,2*H*-perfluorooctyl)-silane (448931, Sigma-Aldrich, USA) under vacuum for 1 hour.

### Microfabrication of PDMS scaffolds

PDMS scaffolds with open cleft free of residual membranes were replica-molded from the master mold using a custom-built compression device. PDMS was mixed in a standard ratio of 10:1 base-to-curing agent (Sylgard 184, Dow Corning, USA), degassed at 70 mbar for 30 min, poured on the master mold placed in the custom-built compression, and degassed at 70 mbar for a maximum of 45 min until all air bubbles were removed. Afterward, a 2-mm-thick polymethyl methacrylate lid was placed on the top at a slight angle, preventing the formation of any air bubbles. Last, the top piece of the compression device was mounted, and all four screws were tightened in a crossed manner with a maximum torque of 4 Nm. After the PDMS was cured at 80°C overnight, each substrate was cut out with an 18-mm-diameter punch, cleaned in 70% ethanol ultrasonic bath for 20 min, and stored in 70% ethanol.

### Functionalization and mounting of PDMS scaffolds

To stabilize the anchorage of the growing μTissue on the edges of the PDMS scaffold, Fn was covalently bound to the surface of the PDMS scaffolds using a heterobifunctional cross-linker (Sulfo-SANPAH, 1 mg/ml; Thermo Fisher Scientific, USA), as described previously (*43*). Fn was isolated from human plasma, as previously described (*83*). The functionalized substrates were attached with optical glue NOA-61 (Norland Optical adhesive 61, Norland, USA) to an ibidi-treated polymer-bottom dish (μ-Dish 35 mm, no. 81158, ibidi) that had previously been passivated using poly(l-lysine)-graft-poly(ethylene glycol) (PLL-g-PEG, 0.1 mg/ml; SuSoS, Switzerland). After assembling the cell culture system, it was washed once with phosphate-buffered saline (PBS) and ultraviolet-treated for 15 min. Before cell seeding, the surface was equilibrated with α-minimum essential medium (αMEM, Biowest SAS) supplemented with 10% fetal bovine serum at 37°C for at least 1 hour.

### μTissue growth experiments

#### Cell culture

Primary normal dermal human fibroblasts (NHDFs, passage numbers 6 to 10, Lonza) were maintained in αMEM (Biowest SAS, France) supplemented with 10% fetal bovine serum and 1% penicillin-streptomycin and changed every 2 to 3 days. All experiments were conducted at 37°C in a humidified atmosphere with 5% CO_2_. The cells were trypsinized and seeded on the PDMS substrates at a density of 2 × 10^5^ cells per substrate. For μTissue experiments, the cell culture medium was supplemented with 100 μM l-ascorbic acid 2-phosphate sesquimagnesium salt hydrate (A8960-5G, Sigma-Aldrich, USA) and Fn (0.05 mg/ml) in PBS. For interventional experiments, the cell culture medium was further supplemented with 0.1% dimethyl sulfoxide (DMSO) and the following components from day 4 of μTissue growth: inhibition of enzymatic TG2 activity with Z006 (100 μM; Zedira GmbH, Germany), inhibition of TGF-βRI/II with GW788388 (20 μM; 3264, Tocris, UK), inhibition of dual tyrosine kinase of EGFR and HER-2 with lapatinib (1 μM; GW572016, Axon Medchem, USA), inhibition of broad-spectrum MMP with GM6001 (25 μM; ab120845, Abcam, UK), inhibition of MMP-2/-9 with SB-3CT (5 μM; ab141579, Abcam, UK), inhibition of α2β1-integrin with BTT3033 (10 μM; no. 4724, Tocris, UK), guinea pig liver transglutaminase (30 μg/ml; T006, Zedira GmbH, Germany), and inhibition of Fn-collagen interaction with R1R2, as previously described (*5*) (2.5 μM; amino sequence GLNGENQKEPEQGERGEAGPPLSGLSGNNQGRPSLPGLNGENQKEPEQGERGEAGPP, manufactured by GenScript, USA).

To assess the αSMA response on Z006 and GW788388, we performed a TGF-β1 stimulation test on 2D Fn-coated glass. Primary NHDFs (passage numbers 6 to 10, Lonza, Switzerland) were cultured routinely in normal growth medium composed of αMEM (Biowest SAS, France) supplemented with 10% fetal bovine serum and 1% penicillin-streptomycin. Once at optimal confluency, the fibroblasts were trypsinized and seeded separately in triplicate in eight-well Labtek chambers at 4.5 × 10^3^ cells/cm^2^ in normal growth medium. After 24 hours, medium was replaced with and without TGF-β1 (5 ng/ml; 100-21C, PeproTech, USA). Each condition was combined with GW788388 (20 μM; 3264, Tocris, UK), Z006 (100 μM; Zedira GmbH, Germany), and 0.1% DMSO and cultured further for an additional 3 days, including a media change at day 3.

To assess the potential toxicity of the different inhibitor treatments on cell viability and metabolic activity, a WST-1 test (Roche, Switzerland) was performed on primary NHDFs cultured in 2D cell culture. Primary NHDFs (passage numbers 6 to 10, Lonza, Switzerland) were cultured routinely in normal growth medium composed of αMEM (Biowest SAS, France) supplemented with 10% fetal bovine serum and 1% penicillin-streptomycin. Once at confluency, the fibroblasts were trypsinized and seeded separately in triplicate in 96-well plates at 4.5 × 10^3^ cells/cm^2^ in normal growth medium. After 4 hours, the cells were exposed to the following inhibitor treatments: Z006 (100 μM; Zedira GmbH, Germany), GW788388 (20 μM; no. 3264, Tocris, UK), GM6001 (25 μM; ab120845, Abcam, UK), SB-3CT (5 μM; ab141579, Abcam, UK), and BTT3033 (10 μM; No 4724, Tocris, UK). Cells cultured in normal growth medium and in growth medium supplemented with 0.1% DMSO were used as control as all inhibitors were reconstituted in DMSO. After 24 hours, tetrazolium salt–containing WST-1 reagent (1:10; no. 05015944001, Roche, Switzerland) was added to the cells and incubated for 1 hour 30 min at 37°C, 5% CO_2_. During this time, metabolically active cells cleave the tetrazolium salts contained in the WST-1 reagent into formazan, which has a higher absorbance than the tetrazolium salts. For all conditions tested, the absorbance of the medium was then measured with a plate reader (Tecan M200). Absorbance values were further normalized by that of the control with 0.1% DMSO condition and reported in fig. S14.

### Decellularization and reseeding

Samples were decellularized following a modified Cukierman protocol (*45*). Briefly, the μTissues were washed twice with prewarmed Hanks’ balanced salt solution HBSS (+ Ca, + Mg) and once with cold sodium deoxycholate [0.5% (w/v); Sigma-Aldrich, USA] in PBS. Cell membranes were removed during two incubations on ice for 10 min with cold sodium deoxycholate [0.5% (w/v); Sigma-Aldrich, USA] in PBS following the Cukierman protocol (*45*). After washing three times in PBS, samples were stored at 4°C during the preparation of cell suspension for reseeding. For reseeding, primary normal dermal fibroblasts were cultured 3 days before reseeding in αMEM (Biowest SAS, France) supplemented with 10% fetal bovine serum and with and without TGF-β1 (5 ng/ml; 100-21C, PeptroTech, USA) to achieve fibroblast and myofibroblast population, respectively. For tracking the reseeded cells, the cells were stained with cell tracker (10 μM; CellTracker Red CMTPX Dye, Thermo Fisher Scientfic, USA) in serum-free αMEM (Biowest SAS) and insulin-transferin-selin (41400-04, Thermo Fisher Scientific, USA) as serum replacement and incubated at 37°C with 5% CO_2_ for 30 min. The dye-containing solution was then removed by incubating the cells with the serum-free medium for 5 min three times, followed by a wash with PBS. The stained cells were trypsinized and seeded in 2 ml of αMEM (Biowest SAS, France) supplemented with 100 μM l-ascorbic acid 2-phosphate sesquimagnesium salt hydrate (A8960-5G, Sigma-Aldrich, USA) and 1% penicillin-streptomycin onto the prewarmed decellularized matrices at a density of 0.5 Mio cells/ml.

### Fixation and immunofluorescence

After 12 days of total incubation, samples were washed twice with prewarmed HBSS (+ Ca, + Mg) and fixed with 4% paraformaldehyde (PFA) for 15 min. The cell membrane was permeabilized with 1% Triton X-100 (T8787-50ML, Sigma-Aldrich, USA) for 20 min followed by 60 min of incubation in blocking solution [2% bovine serum albumin (BSA) (w/v) (05470-5G, Sigma-Aldrich, USA) and 5% donkey serum (v/v) (ab7475, Abcam, UK)]. μTissues depicted in Figs. 1, 3, 4, and 6 were immunostained against TNC, and μTissues depicted in Fig. 5 were immunostained against TG2. Monoclonal TNC antibody (1:100; BC-24, MA1-26779, Thermo Fisher Scientific, USA) was combined with Fn (C-20) antibody (1:100; sc6952, Santa Cruz Biotechnology, USA) and were supplemented in blocking solution to the respective μTissues and incubated for 1 hour at RT. After washing three times in PBS for 5 min each, samples were incubated with anti-goat Alexa Fluor 488 (1:200; ab260129, Abcam, UK) and anti-mouse Alexa Fluor 647 (1:100; ab150107, Abcam, UK) in blocking solution for 1 hour at RT. After three additional PBS washes, αSMA was stained with Alexa Fluor 594–conjugated anti-αSMA antibody (1:100; ab202368, Abcam) in blocking solution for 1 hour at RT followed by three PBS washes. In addition to the described staining protocol, we tested different TNC antibodies in μTissues depicted in fig. S8. As TNC has a variety of epitopes that might be masked upon ECM binding, we confirmed the existence of a steep TNC gradient between the μTissue growth front and core regions with different antibodies as various TNC splice variants might exist. In addition to the monoclonal antibody BC-24 (epitope EGF repeats; MA1-26779, Thermo Fisher Scientific, USA), which was used as standard throughout the study, we also used two polyclonal TNC antibodies and a second monoclonal TNC B28.13 antibody, confirming that TNC mainly accumulated within the early growth region and thus defines immature regions of the μTissues (fig. S11). We tested different epitopes by using a polyclonal TNC antibody [produced in rabbit; (*84*)] counterstained with anti-rabbit Alexa Fluor 555 (1:100; A21429, Thermo Fisher Scientific) and another monoclonal TNC B28.13 [epitope constant Fn-III repeat 6 or 7 (*51*), produced in-house] counterstained with anti-mouse Alexa Fluor 647 (1:100; ab150107).

In the samples shown in Fig. 5A, primary TG2 antibody (1:100; CUB 7402, ab2386, Abcam, UK) was supplemented in blocking solution, incubated for 1 hour at RT, and, after washing three times in PBS for 5 min each, counterstained with anti-mouse Alexa Fluor 647 (1:100; ab150107, Abcam, UK). After washing three times in PBS for 5 min each, αSMA was stained with Alexa Fluor 594–conjugated anti-αSMA antibody (1:100; ab202368, Abcam, UK) in blocking solution for 1 hour at RT followed by three PBS washes, following the above protocol. Staining of samples in Fig. 5 (B and C) followed the same protocol with the addition of Fn (C-20) antibody (1:100; sc6952, Santa Cruz Biotechnology, USA) counterstained with anti-goat Alexa Fluor 488 (1:200; ab260129, Abcam, USA).

Fn fiber tension probing in Fig. 2B was done using the Fn binding peptide FnBPA5. FnBPA5-Cy5 was commercially synthesized and high-performance liquid chromatography–purified, as described previously (*82*). After 12 days of total incubation, the μTissues depicted in Fig. 2B were washed twice with prewarmed HBSS (+ Ca, + Mg) and further incubated for 1 hour at 37°C with FnBPA5-Cy5 (5 μg/ml) diluted in HBSS (+ Ca, + Mg). The samples were then washed three times with prewarmed HBSS (+ Ca, + Mg) before being fixed with 4% PFA for 15 min and blocked for 60 min with a blocking solution [2% BSA (w/v) (05470-5G, Sigma-Aldrich, USA, and 5% donkey serum (v/v) (ab7475, Abcam, UK)]. Immunostaining against Fn was further performed following the protocol mentioned above with Fn (C-20) antibody (1:100; sc6952, Santa Cruz Biotechnology) counterstained with anti-goat Alexa Fluor 488 (1:200; ab260129, Abcam, UK).

The reseeded μTissues in Fig. 2 were fixed after 13 days of total incubation (24 hours after reseeding), permeabilized and blocked as described above, and, afterward, stained with Alexa Fluor 594–conjugated anti-αSMA antibody (1:100; ab202368, Abcam, UK) in blocking solution for 1 hour at RT followed by three PBS washes.

### Microscopy and image analysis

#### Image acquisition

Phase-contrast images of the μTissues were acquired using a Zeiss Axiovert 200 M inverted microscope with 5× objective. Confocal fluorescence imaging was performed using a Leica TCS SP8 MP inverted multiphoton laser scanning microscope with a 25× objective (0.95NA L Water HCX IRAPO). For FnBPA5 (Fig. 2B) and reseeding experiments (Fig. 2, D and E), a 10× objective (0.3NA HC PL FLUOTAR) was used. Fluorophores were directly excited in single-photon mode. Mature collagen fibers were imaged by SHG in multiphoton laser scanning mode with an open pinhole. Second harmonic was generated with a Mai Tai XF (Spectra-Physics, USA) femtosecond Ti-sapphire–pulsed laser tuned at 880 nm, and signal emitted from collagen fibers was detected at 440 nm.

### Image analysis

#### Growth distances

Growth distances were measured between the angle tip of the cleft and the μTissue-medium interphase along the bisector angle axis in the phase-contrast images of the respective time points.

#### Visualization and profile intensity plots

All images were rotated such that the angle tip of the cleft is oriented left-handed, and the bisector angle of the cleft is parallel to the *x* axis of the image. A representative symmetry axis parallel to the *x* axis and in close proximity to the bisector angle was visually assessed such that it represents the young-to-mature gradient in each μTissue. Profile intensities were assessed along this symmetry axis by the column intensity average of 100 pixels equally distributed around the symmetry axis at each *x* position. Profile intensity data used for plots were normalized by the respective maximum level of each channel, which allows relative comparison on an interchannel and intertissue level. For orientation and comparison of the absolute intensities, the integral of each raw intensity profile is plotted in fig. S6 (B to H). To determine the distance to growth front (e.g., Fig. 1, K and L), all channels were cumulated, and the boundaries of the μTissue were defined at the *x* position, where cumulative signal intensity was greater than the threshold of 25% of maximum intensity at the tissue-medium interphase. The overview visualizations with split look-up tables were created by a custom-built ImageJ (National Institutes of Health, USA) script and split at the *y* position of the earlier determined symmetry axis used for profile plot. For visualization, the contrast of confocal and two-photon laser scanning images was linearly adjusted to saturate 0.05% of all pixels; this allows visualization of tissue morphology and relative intensity comparison between the growth front and the core region of one tissue. The absolute gray values of the original images, without contrast adjustment, are depicted as integrated intensities of the respective profile plots in fig. S6 (B to H).

### Fiber orientation

Fiber orientation analysis was carried out using a custom-written MATLAB script. Orientation of the fibrous structure found in the SHG images was analyzed on the basis of a filtering method using a custom-written MATLAB script (2020a, Mathworks, MA, USA). We here used a 5 × 5 basic operator *H*_x_ optimized by Kroon with *H*_y_ being the transposition of *H*_x_

$$H_{x}=\left(\begin{array}{ccccc} 0.0007 & 0.0052 & 0.0370 & 0.0052 & 0.0007\\ 0.0037 & 0.1187 & 0.2589 & 0.1187 & 0.0037\\ 0 & 0 & 0 & 0 & 0\\ -0.0037 & -0.1187 & -0.2589 & -0.1187 & -0.0037\\ -0.0007 & -0.0052 & -0.0370 & -0.0052 & -0.0007 \end{array}\right)$$ (1)

*H*_xx_ = *H*_x_·*H*_x_, *H*_yy_ = *H*_y_·*H*_y_, and *H*_xy_ *= H*_x_·*H*_y_, where the dot product represents a sequential application of the basic operators to the image, were applied to the preprocessed SHG images to obtain the second-order image derivatives *I*_xx_, *I*_yy_, and *I*_xy_, respectively. These outcomes were processed using a Gaussian filter with 4 pixel sigma, resulting in smoothened image derivatives I^*^_xx_, I^*^_yy_, and I^*^_xy_. The pixel-level local orientation ϕ was then computed as follows

$$\varphi (i,j)=0.5\arctan\frac{-2{I^{*}}_{\mathrm{xy}}(i,j)}{{I^{*}}_{\mathrm{xx}}(i,j)-{I^{*}}_{\mathrm{yy}}(i,j)}$$ (2)

The fiber orientation was visualized in HSV color space, where the hue and value of each pixel are 2ϕ and the brightness, respectively. Saturation is set to 1. Note that the brightness was optimized by linearly adjusting the contrast of the original image using the “imadjust” function of MATLAB for visualization.

### Statistical analysis

Statistical testing, as indicated in each figure caption, was performed using GraphPad Prism (version 9.2.0, GraphPad Software LCC, USA). A *P* value less than 0.05 was considered statistically significant. Error bars represent 95% confidence intervals, unless indicated differently.

## Notes

View/request a protocol for this paper from *Bio-protocol*.
