# Supplementary material for: How the mechanobiology orchestrates the iterative and reciprocal ECM-cell cross-talk that drives microtissue growth
Source: Sci Adv. 2023 Mar 29;9(13):eadd9275. doi: 10.1126/sciadv.add9275 (PMC10058249; doi:10.1126/sciadv.add9275)
Supplement: Supplementary file 1 — Figs. S1 to S14 Legend for data S1 Legends for movies S1 and S2 [file sciadv.add9275_sm.pdf]

Science Advances
AAAS

Supplementary Materials for

How the mechanobiology orchestrates the iterative and reciprocal ECM-cell cross-talk that drives microtissue growth

Mario C. Benn *et al.*

Corresponding author: Viola Vogel, viola.vogel@hest.ethz.ch; Mario C. Benn, mario.benn@hest.ethz.ch

Sci. Adv. **9**, eadd9275 (2023)
DOI: 10.1126/sciadv.add9275

The PDF file includes:

Figs. S1 to S14
Legend for data S1
Legends for movies S1 and S2

Other Supplementary Material for this manuscript includes the following:

Data S1
Movies S1 and S2

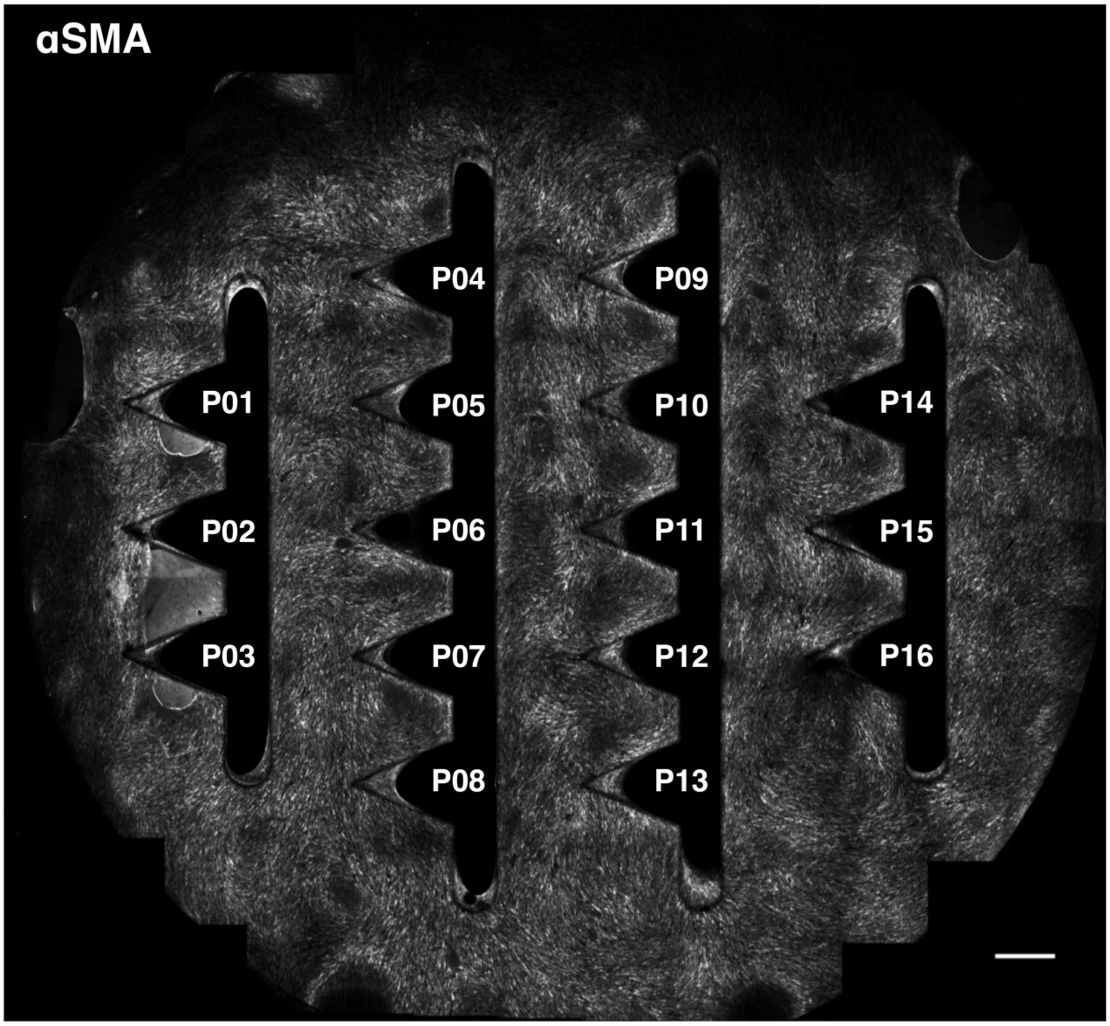

Fig. S01. Overview of full PDMS cleft scaffold array after 12 days incubation stitched together from tile scans. While the scaffold was seeded with fibroblasts, αSMA expressing myofibroblasts appear only in the growth front layer that surrounds the µTissues which grow inside 16 different cleft positions (P01 – P16). Note: the focal plane of the top layer and of each cleft µTissue are mismatched due to the different refractive indices of PDMS and PBS. Scale bar 1 mm.

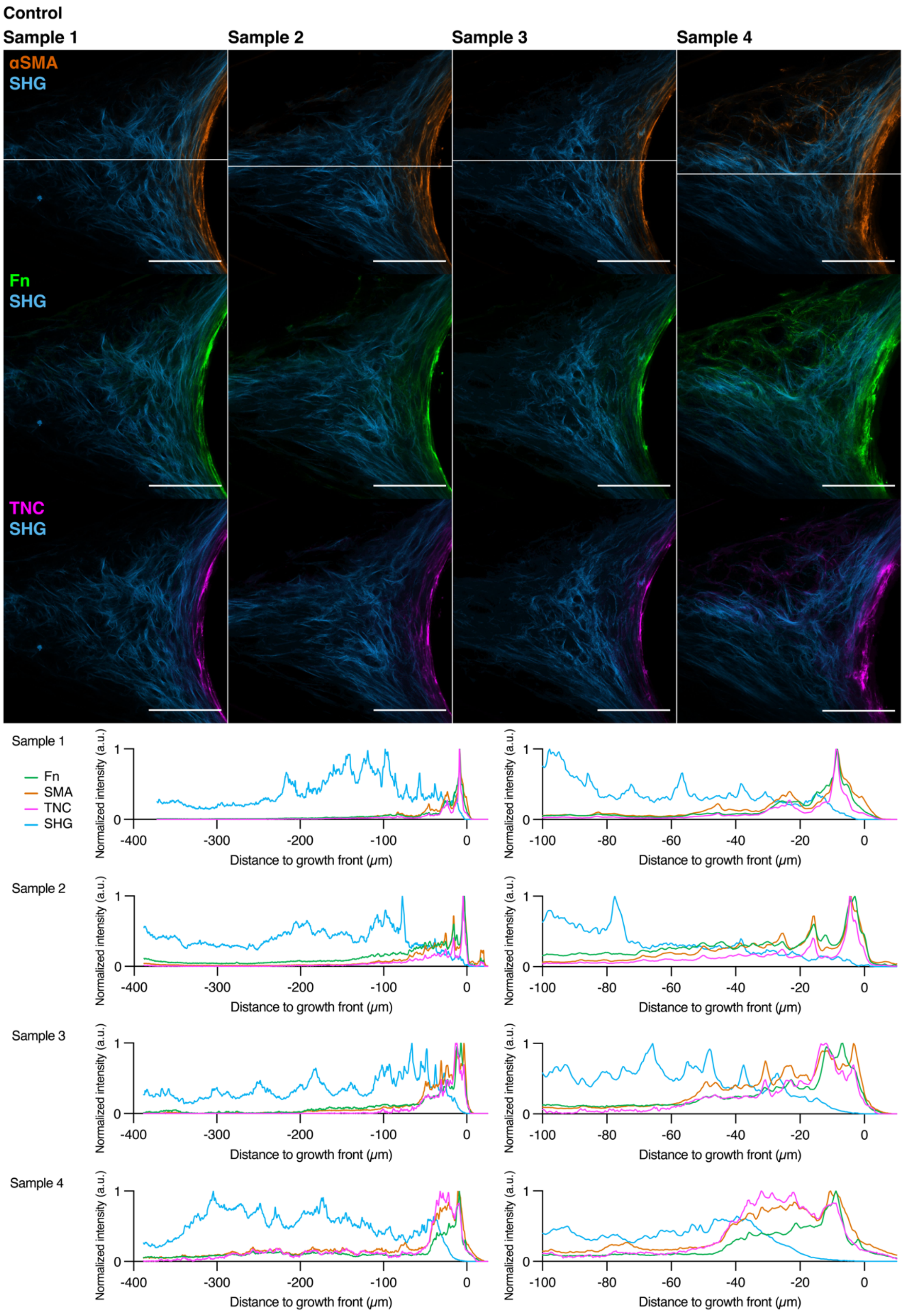

Control
Sample 1
Sample 2
Sample 3
Sample 4
αSMA
SHG
Fn
SHG
TNC
SHG
Sample 1
Sample 2
Sample 3
Sample 4
Fn
SMA
TNC
SHG
Normalized intensity (a.u.)
Distance to growth front (µm)

Fig. S02. Superimposed confocal and 2-photon laser scanning microscopy (CLSM+SHG) images and intensity profiles of four different control µTissues. The images were taken at the midplane of the µTissues. Data acquired from 12 days old µTissues, after control interventions were performed with DMSO starting at day 4 (see also Fig. 1H-L and Fig. 2A+E). Upper part: Superimposed central ROIs of representative control µTissues (DMSO, Sample 1–4) imaged with confocal and 2-photon laser scanning microscopy (CLSM+SHG) for αSMA (orange, upper row), Fn (green, middle row), TNC (magenta, lower row) and SHG (blue, all rows). Scale bar 100 µm. Lower part: normalized intensity profiles of the respective channels Fn, αSMA, TNC and SHG, which are quantified along the cleft's bisector angle (white lines in upper αSMA panels) over the distance from the growth front. An enlarged plot of the first 100 µm distance from the growth front is presented on the right side. The mean of 100 vertically accumulated pixels (50px above and 50px underneath the white line) is plotted at each distance from the growth front. To determine the distance from the growth front, all channels were cumulated and the boundaries of the µTissue were defined at the x position where the cumulative signal intensity was greater than a 25% maximum-intensity threshold at the tissue-medium interphase. The absolute grey values are depicted as integrated intensities of the respective profile plots in Fig. S06C.

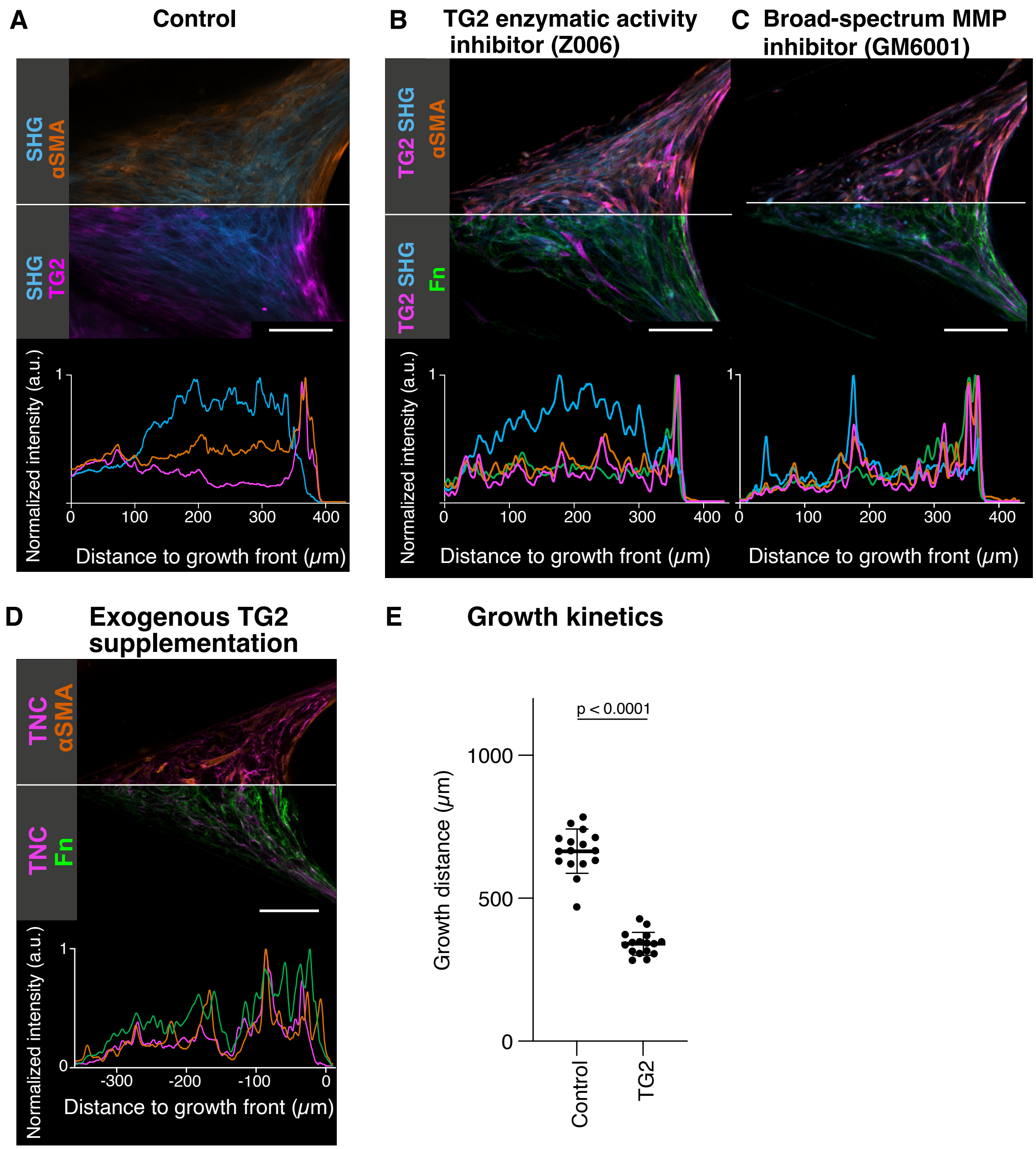

Fig. S03. TG2 distribution superimposed with other µTissue markers in the presence of either inhibitors or exogenous TG2 supplementation. The images were taken at the midplane of the µTissues. (**A** to **C**) TG2 stain of 12 days old µTissues, after interventions with various inhibitors at day 4. Upper row: Representative ROIs of µTissues imaged with confocal and 2-photon laser scanning microscope (CLSM+SHG): (**A**) control (DMSO), (**B**) specific inhibition of enzymatic TG2 activity (Z006), and (**C**) broad-spectrum MMP inhibition (GM6001). The CLSM+SHG data are depicted with two different sets of look up table; top: αSMA (orange),

SHG (cyan); bottom: TG2 (magenta), Fn (green), SHG (cyan). Scale bars 100 µm. See also Fig. S13 for more samples. Lower row: Respective normalized intensity profiles of the various channels along the cleft bisector angle. The mean of 100 vertically accumulated pixels (50px above and 50px underneath the white line) is plotted at each distance from the growth front. (**D**) µTissue supplemented with exogenous TG2 at day 5. Upper part: Superimposed confocal laser scanning microscopy (CLSM+SHG) images depicted with two different sets of look up table; top: αSMA (orange), TNC (magenta); bottom: TNC (magenta), Fn (green), SHG (cyan). Scale bar 100 µm. Lower part: Normalized intensity profile of the respective channels Fn, αSMA, and TNC as plotted along the cleft bisector angle (white line) over the distance from the growth front. (**E**) Growth kinetics of control µTissues versus µTissues treated with exogenously supplemented TG2 starting at day 4, visualized as growth distances (µm) at day 12. Growth distances were measured on phase-contrast images between the tip of the angular cleft and the µTissue-medium interphase along the bisector angle axis (see Fig. 1F). A Student's *t*-test was used to test the significance of the difference between these two groups.

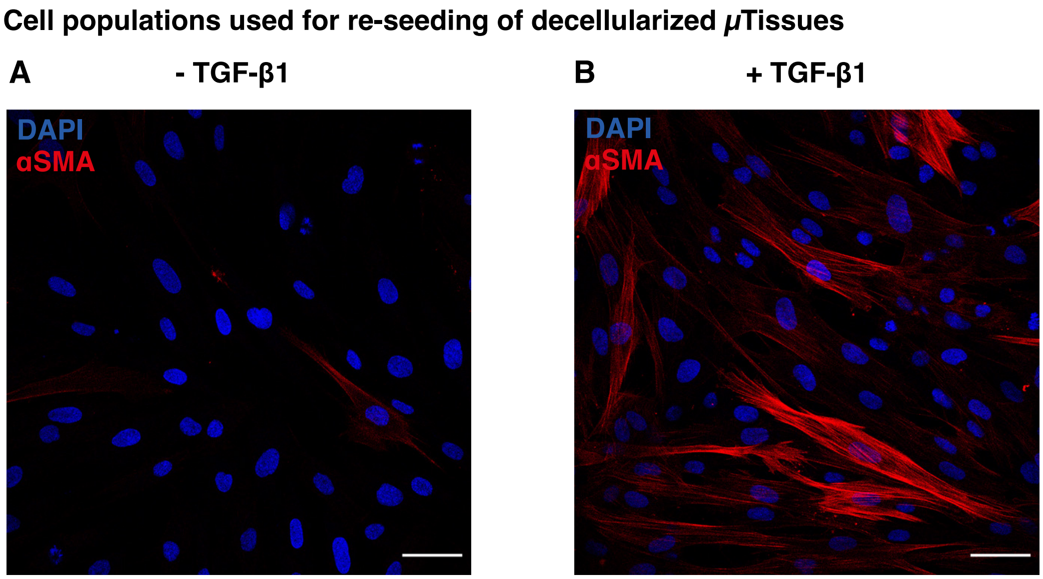

Fig. S04. Fibroblasts stimulated with exogenously supplemented TGF-β1 in 2D cell culture and subsequently used for the re-seeding experiments on decellularized μTissues. Fibroblasts were cultivated on fibronectin (Fn) coated glass without (A) and with TGF-β1 supplementation (B) for 72 hrs prior to re-seeding decellularized μTissues, as shown in Fig. 2D+E. At the time of re-seeding on the decellularized μTissues, the respective populations were seeded on 2D substrates in parallel, and stained for αSMA and DAPI. Confocal laser scanning images superimposed with DAPI (blue) and αSMA (red) signal are shown. Scale bars 10 μm.

EGFR/HER-2 inhibitor (Lapatanib)

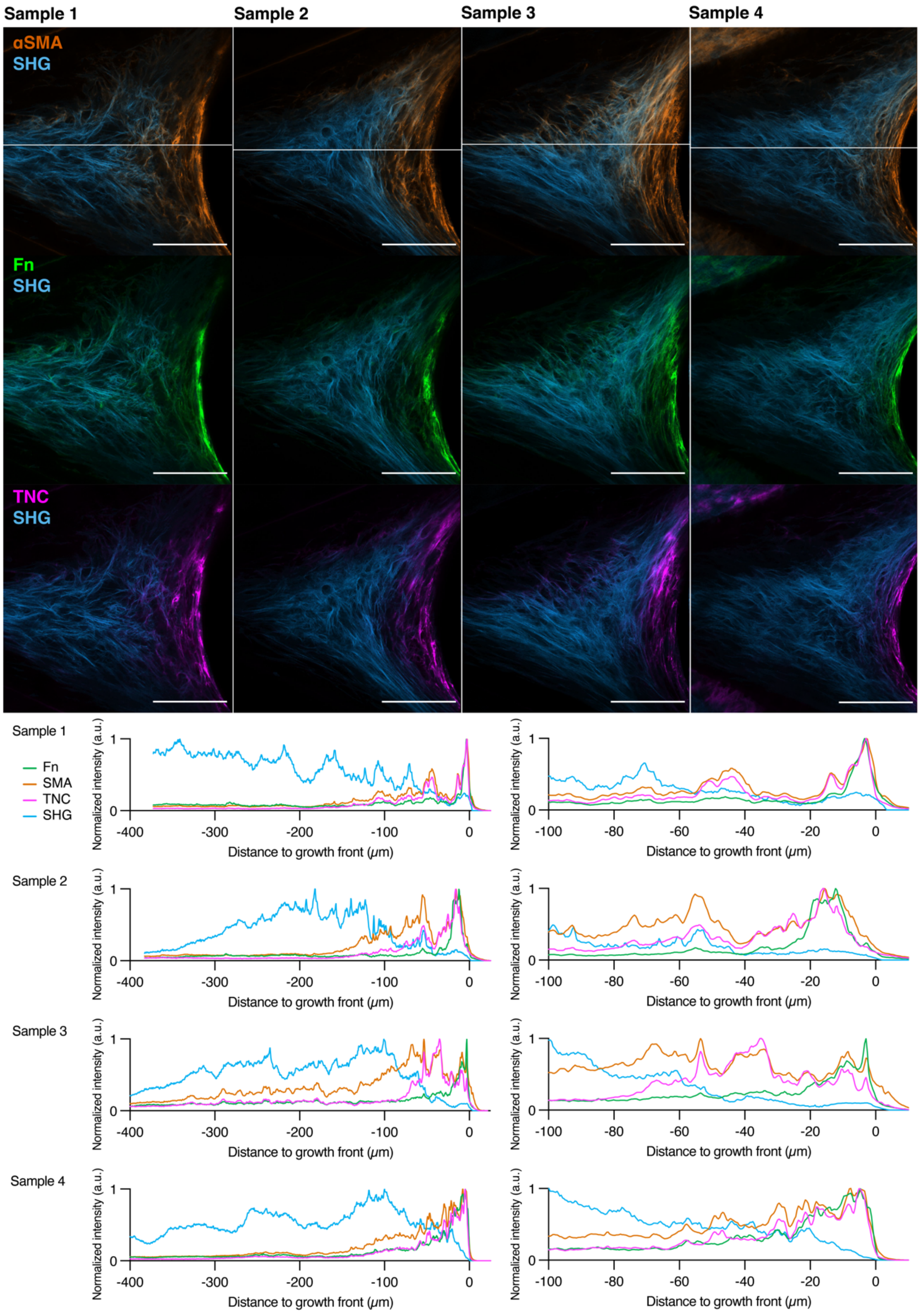

Fig. S05. Superimposed confocal and 2-photon laser scanning microscopy (CLSM+SHG) images and intensity profiles of various µTissues treated with EGFR/HER-2 signaling inhibitor (Lapatinib). The images were taken at the midplane of the µTissues. Data acquired from 12 days old µTissues after interventions with epidermal growth factor receptor (EGFR) and human EGFR-2 (HER-2) signaling inhibitor (Lapatinib) starting at day 4 (see also Fig. 2B+F). Upper part: Superimposed central ROIs of representative µTissues (Sample 1–4) and imaged with confocal and 2-photon laser scanning microscopy (CLSM+SHG) for αSMA (orange, upper row), Fn (green, middle row), TNC (magenta, lower row) and SHG (blue, all rows). Scale bar 100 µm. Lower part: normalized intensity profiles of the respective channels Fn, αSMA, TNC and SHG, which are plotted along the cleft bisector angle (white lines in upper αSMA panels) over the distance from the growth front. An enlarged plot of the first 100 µm distance from the growth front is presented on the right side. The mean of 100 vertically accumulated pixels (50px above and 50px underneath the white line) is plotted at each distance from the growth front. To determine the distance from the growth front, all channels were cumulated and the boundaries of the µTissue were defined at the x position where the cumulative signal intensity was greater than a 25% maximum-intensity threshold at the tissue-medium interphase. The absolute grey values are depicted as integrated intensities of the respective profile plots in Fig. S06C.

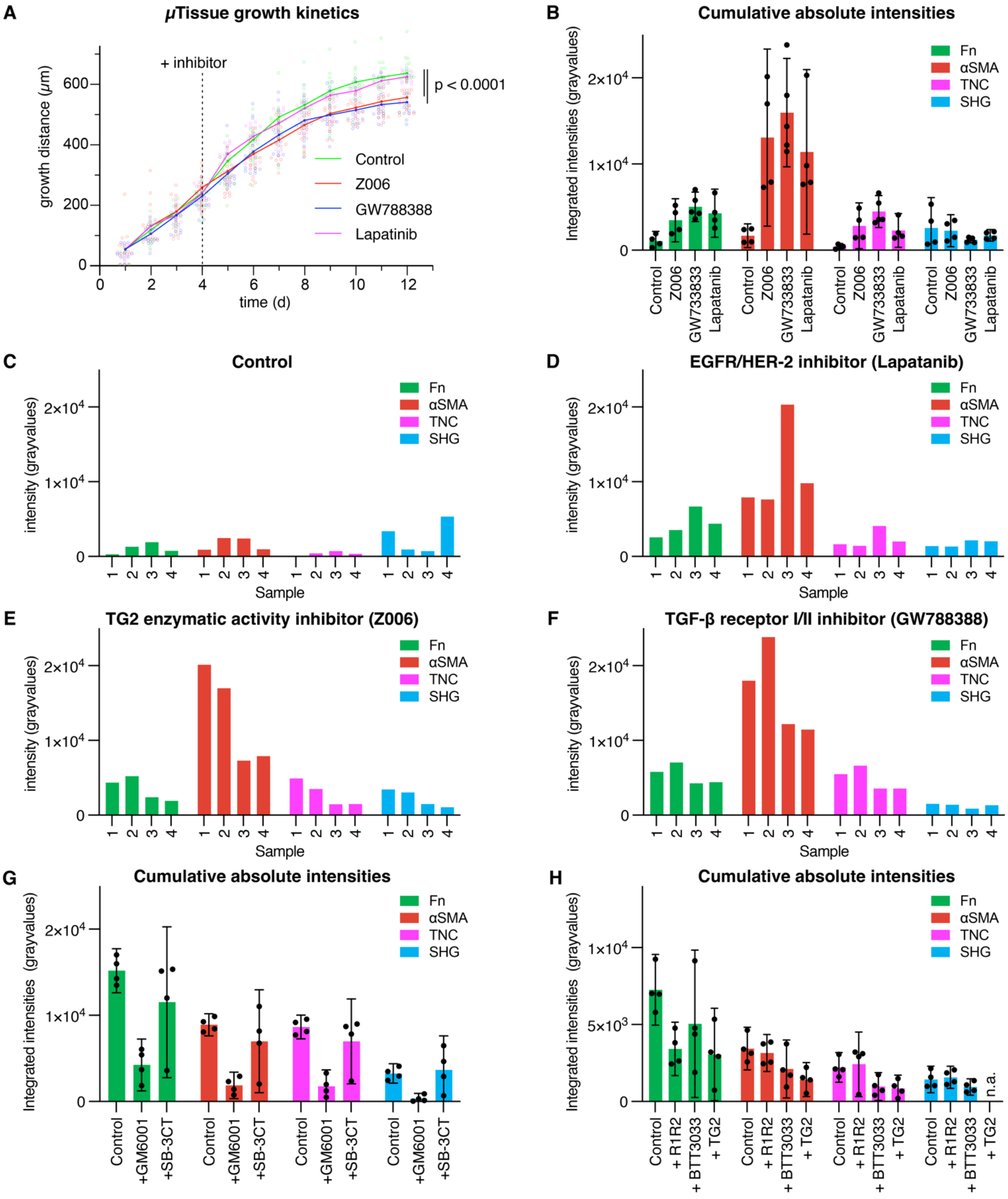

A
μTissue growth kinetics
growth distance (μm)
+ inhibitor
p < 0.0001
Control
Z006
GW788388
Lapatinib
time (d)
B
Cumulative absolute intensities
Integrated intensities (grayvalues)
Fn
αSMA
TNC
SHG
Control
Z006
GW733833
Lapatinib
C
Control
intensity (grayvalues)
Sample
D
EGFR/HER-2 inhibitor (Lapatanib)
E
TG2 enzymatic activity inhibitor (Z006)
F
TGF-β receptor I/II inhibitor (GW788388)
G
Cumulative absolute intensities
Control
+GM6001
+SB-3CT
H
Cumulative absolute intensities
Control
+ R1R2
+ BTT3033
+ TG2
n.a.

Fig. S06. µTissue growth kinetics and fluorescence signal quantifications. (**A**) µTissue growth data as quantified from phase-contrast images (n=20) from control µTissues (DMSO, Fig. 3A and S02), and upon epidermal growth factor receptor (EGFR) inhibition, or human EGFR-2 (HER-2) signaling inhibition (Lapatinib, Fig. 3B and S05), or specific enzymatic TG2 activity inhibition (Z006, Fig. 3C and Fig. S10), or TGF-β receptor signaling inhibition (GW788388, Fig. 3D and Fig. S11). These µTissues were imaged every 24 hours with phase-contrast microscopy and the growth distance between growth front and cleft corner was quantified. Growth distances were measured on phase-contrast images between the tip of the angular cleft and the µTissue-medium interphase along the bisector angle axis (see Fig. 1F). Inhibitor supplementation occurred after 5 days of incubation. The significance of differences in growth distance at day 12 was tested by many-to-one comparisons using Dunn's test (vs Control). (**B**-**H**) The absolute grey values of the original images are depicted as integrated intensities of the respective profile plots. (**B**) Means (n=4) with 95% CI. (**C**) Integrated intensities for all representative samples for control µTissues (DMSO, Fig. 3A and S02), and following (**D**) EGFR/HER-2 signaling inhibition (Lapatinib, Fig. 3B and S05), (**E**) specific enzymatic TG2 activity inhibition (Z006, Fig. 3C and Fig. S10), and (**F**) TGF-β receptor signaling inhibition (GW788388, Fig. 3D and Fig. S11). (**G**) Integrated intensities of the respective profile plots of µTissues treated with broad-spectrum MMP inhibitor (GM6001, Fig. 4B and S12A) and the selective MMP-2 and MMP-9 inhibitor (SB3CT, Fig. 4C and S12B). Mean (n=4) with 95% CI. (**H**) Integrated intensities of the respective profile plots of µTissues treated with a collagen-derived peptide that partially inhibits Fn-collagen interactions (R1R2, Fig. 4D and S12D), an α2β1-integrin inhibitor (BTT3033, Fig. 4E and S12C) or supplementation of exogenous TG2 (Fig. S03D and S12E). Mean (n=4) with 95% CI.

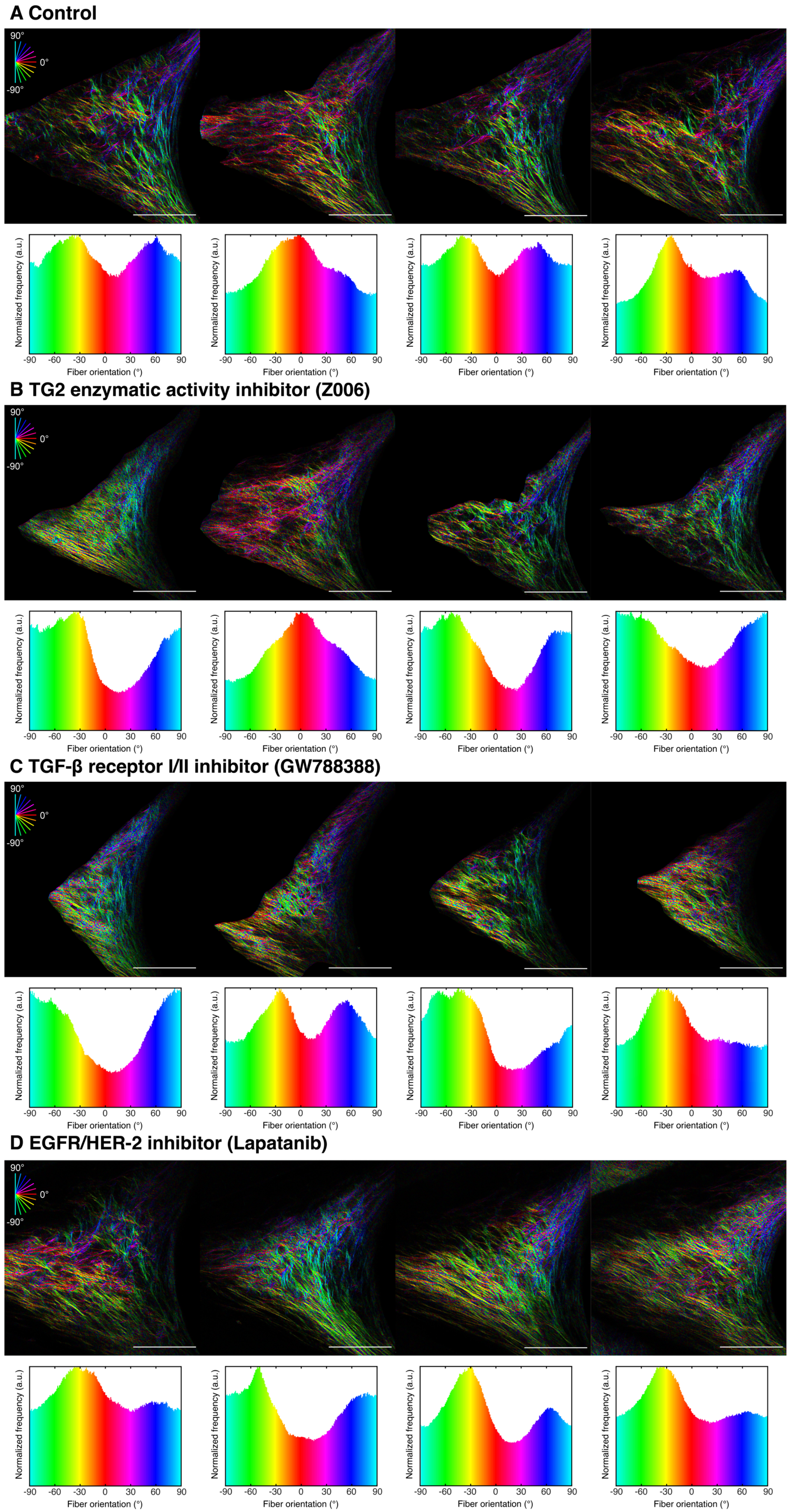
A Control
90°
0°
-90°
Normalized frequency (a.u.)
-90 -60 -30 0 30 60 90
Fiber orientation (°)
B TG2 enzymatic activity inhibitor (Z006)
90°
0°
-90°
Normalized frequency (a.u.)
-90 -60 -30 0 30 60 90
Fiber orientation (°)
C TGF-β receptor I/II inhibitor (GW788388)
90°
0°
-90°
Normalized frequency (a.u.)
-90 -60 -30 0 30 60 90
Fiber orientation (°)
D EGFR/HER-2 inhibitor (Lapatanib)
90°
0°
-90°
Normalized frequency (a.u.)
-90 -60 -30 0 30 60 90
Fiber orientation (°)

Fig. S07. Analysis of collagen (SHG) fiber orientations within four representative µTissues following various inhibitor treatments. Collagen fiber (SHG) orientations depicted as pseudo-coloured ROI for representative µTissues. Fiber orientation analysis was carried out using a custom-written MATLAB script for representative µTissues: (**A**) control, (**B**) enzymatic TG2 activity inhibition (Z006, Fig. 3C and Fig. S10), (**C**) TGF-β receptor signaling inhibition (GW788388, Fig. 3D and Fig. S11), and (**D**) epidermal growth factor receptor (EGFR) and human EGFR-2 (HER-2) signaling inhibition (Lapatinib, Fig. 3B and S05). Upper rows: Collagen fiber (SHG) orientations depicted as pseudo-colored ROIs, color-coded from cyan (± 90°) to red (0°). Scale bar 100 µm. Lower rows: normalized collagen fiber (SHG) orientation frequency distributions depicted as histograms.

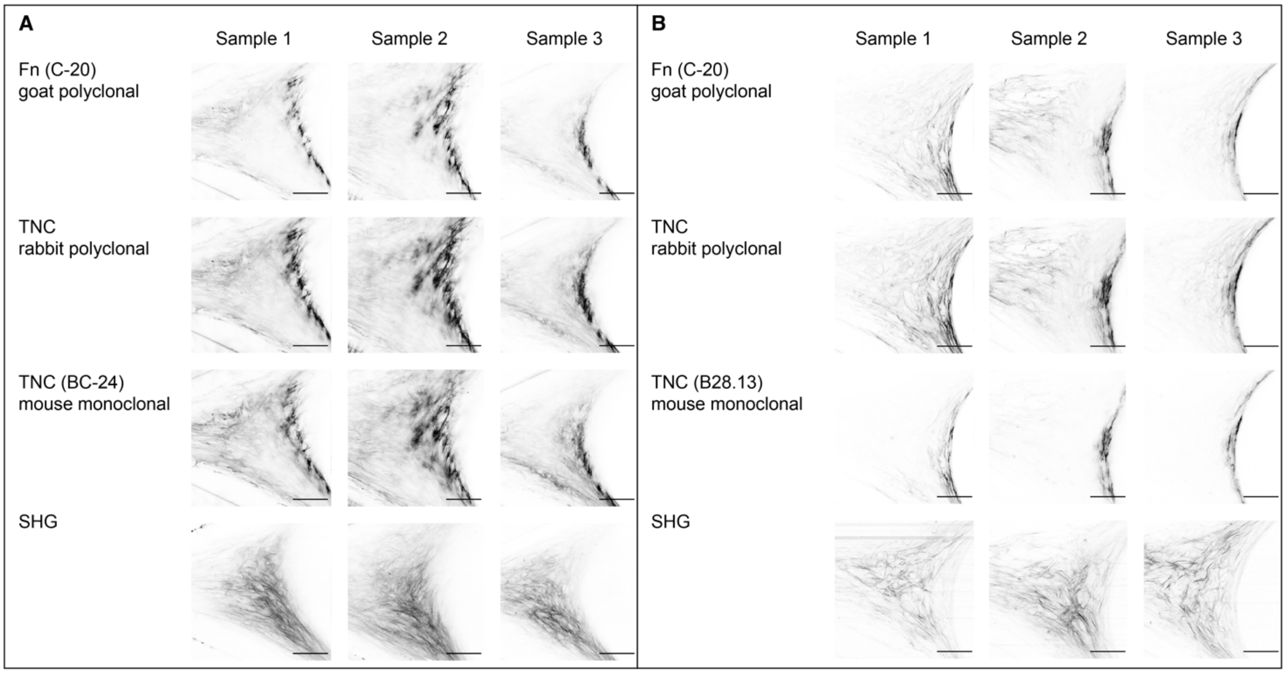

Fig. S08. Supplementary tenascin-C (TNC) epitope investigations. Data acquired with confocal and 2-photon laser microscopy from 12 days old µTissues. The images were taken at the midplane of the µTissues. Depicted are grey scaled immunofluorescence images of each µTissue (Sample 1-3) with their respective fibronectin (Fn) and second harmonic generation (SHG) channels. To ensure that TNC detection is not masked due to cryptic epitopes within the interior of the µTissues, we examined µTissues stained with a rabbit polyclonal TNC antibody (*84*), (**A**) the mouse monoclonal antibody BC-24 (epitope EGF repeats; MA1-26779, ThermoFisher), which is used as standard throughout the study, and (**B**) a second mouse monoclonal antibody TNC B28.13 (epitope constant Fn-III repeat 6 or 7 (*51*), produced in house). Scale bar 100 µm.

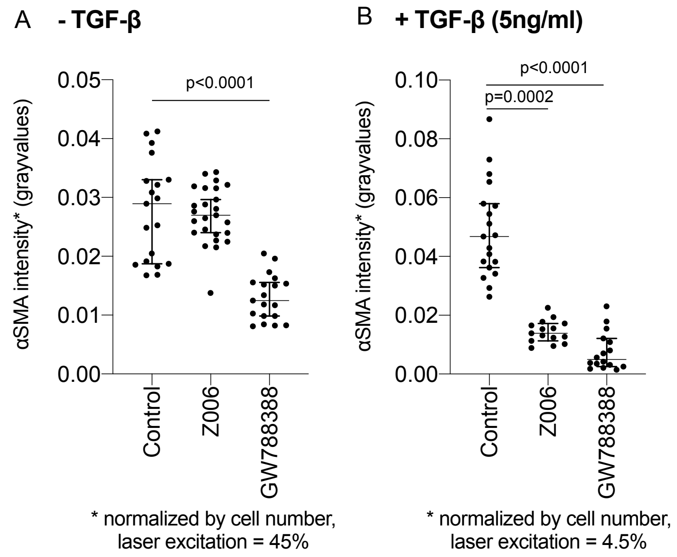

Fig. S09. TGF-β1 stimulation in 2D cell culture without or with either Z006 or GW733833 treatment. Inhibitor test on planar glass substrates coated with adsorbed Fn for one hour prior to seeding of normal human dermal fibroblasts. TGF-β1 and inhibitors were supplemented 24hrs after cell seeding, medium was exchange with fresh supplements every 2 days. Cells were fixed with 4% PFA and stained 4 days after TGF-β1 and inhibitor treatment was started. αSMA intensities were normalized by the cell number. Cell numbers were determined with cell counter plugin for ImageJ (NIH, USA) based on DAPI staining. The significance of differences was tested by performing many-to-one comparisons using Dunn's test (vs Control).

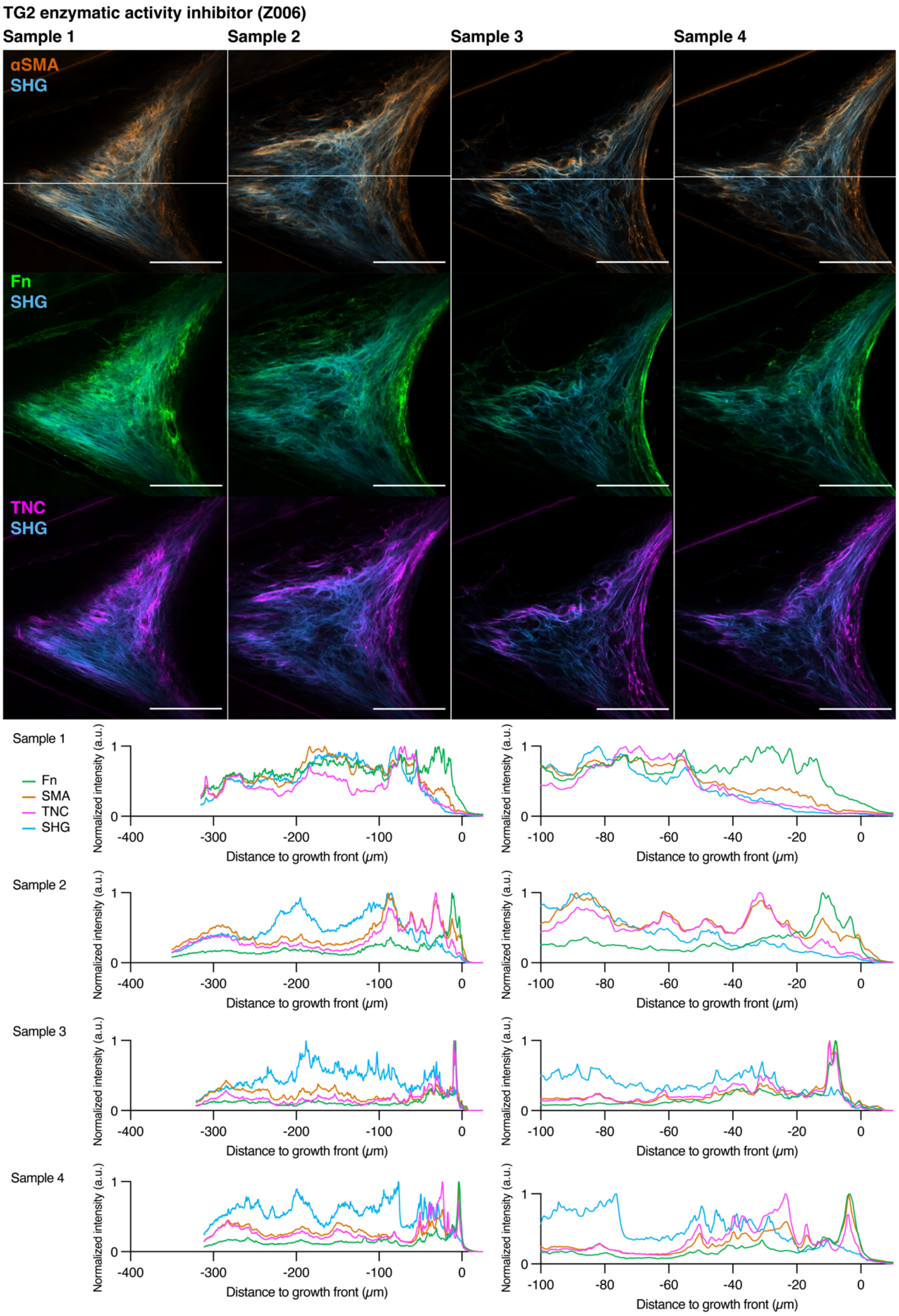
TG2 enzymatic activity inhibitor (Z006)
Sample 1
Sample 2
Sample 3
Sample 4
αSMA
SHG
Fn
SHG
TNC
SHG
Sample 1
Sample 2
Sample 3
Sample 4
Fn
SMA
TNC
SHG
Normalized intensity (a.u.)
Distance to growth front (μm)
-400
-300
-200
-100
0
-80
-60
-40
-20
1

Fig. S10. Superimposed confocal and 2-photon laser scanning microscopy (CLSM+SHG) images and intensity profiles of four representative µTissues treated with TG2 enzymatic activity inhibitor Z006. The images were taken at the midplane of the µTissues. Data acquired from 12 days old µTissues after interventions with TG2 enzymatic activity inhibitor (Z006) starting at day 4 (see also Fig. 2C+G). Upper part: Superimposed central ROIs of µTissues Samples 1–4 and imaged with confocal and 2-photon laser scanning microscopy (CLSM+SHG) for αSMA (orange, upper row), Fn (green, middle row), TNC (magenta, lower row) and SHG (blue, all rows). Scale bar 100 µm. Lower part: normalized intensity profiles of the respective channels Fn, αSMA, TNC and SHG, which are plotted along the cleft bisector angle (white lines in upper αSMA panels) over the distance from the growth front to the cleft corner. An enlarged plot of the first 100 µm distance from the growth front is presented on the right side. The mean of 100 vertically accumulated pixels (50px above and 50px underneath the white line) is plotted at each distance from the growth front. To determine the distance from the growth front, all channels were cumulated and the boundaries of the µTissues were defined at the x position where the cumulative signal intensity was greater than the a 25% maximum-intensity threshold at the tissue-medium interphase. The absolute grey values are depicted as integrated intensities of the respective profile plots in Fig. S06C.

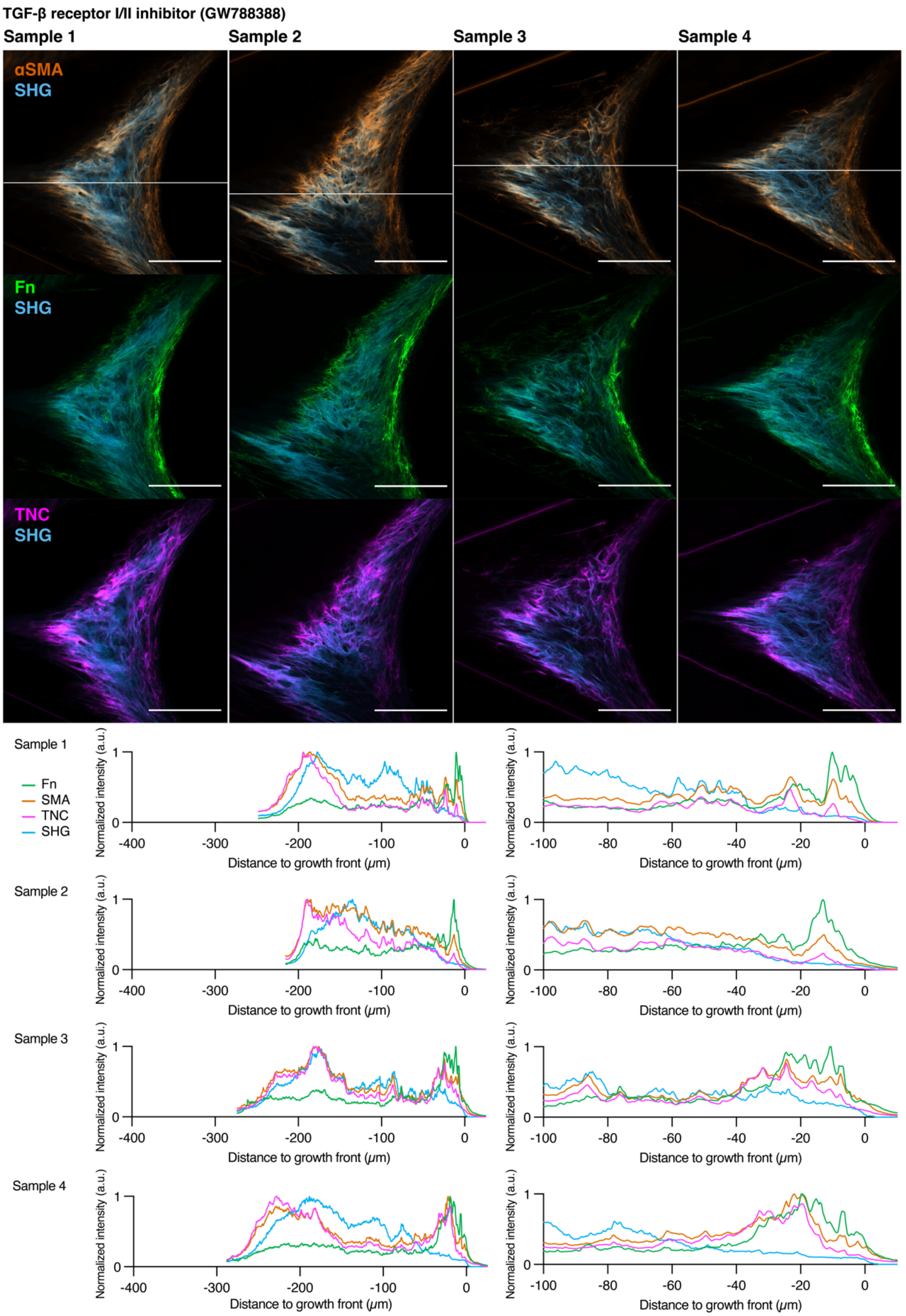
TGF-β receptor I/II inhibitor (GW788388)
Sample 1
Sample 2
Sample 3
Sample 4
αSMA
SHG
Fn
SHG
TNC
SHG
Sample 1
Sample 2
Sample 3
Sample 4
Fn
SMA
TNC
SHG
Normalized intensity (a.u.)
Distance to growth front (μm)

Fig. S11. Superimposed confocal and 2-photon laser scanning microscopy (CLSM+SHG) images and intensity profiles of four representative µTissues treated with the TGF-β receptor I/II inhibitor GW788388. The images were taken at the midplane of the µTissues. Data acquired from 12 days old µTissues after intervention with TGF-β receptor I/II inhibitor GW788388 starting at day 4 (see also Fig. 2D+H). Upper part: Superimposed central ROIs of representative µTissues (Sample 1–4) and imaged with confocal and 2-photon laser scanning microscopy (CLSM+SHG) for αSMA (orange, upper row), Fn (green, middle row), TNC (magenta, lower row) and SHG (blue, all rows), respectively. Scale bar 100 µm. Lower part: normalized intensity profiles of the respective channels Fn, αSMA, TNC and SHG, which are plotted along the cleft bisector angle (white lines in upper αSMA panels) over the distance from the growth front. An enlarged plot of the first 100 µm distance from the growth front is presented on the right side. The mean of 100 vertically accumulated pixels (50px above and 50px underneath the white line) is plotted at each distance from the growth front. To determine the distance from the growth front, all channels were cumulated and the boundaries of the µTissue were defined at the x position where the cumulative signal intensity was greater than a 25% maximum-intensity threshold at the tissue-medium interphase. The absolute grey scale values are depicted as integrated intensities of the respective profile plots in Fig. S06C.

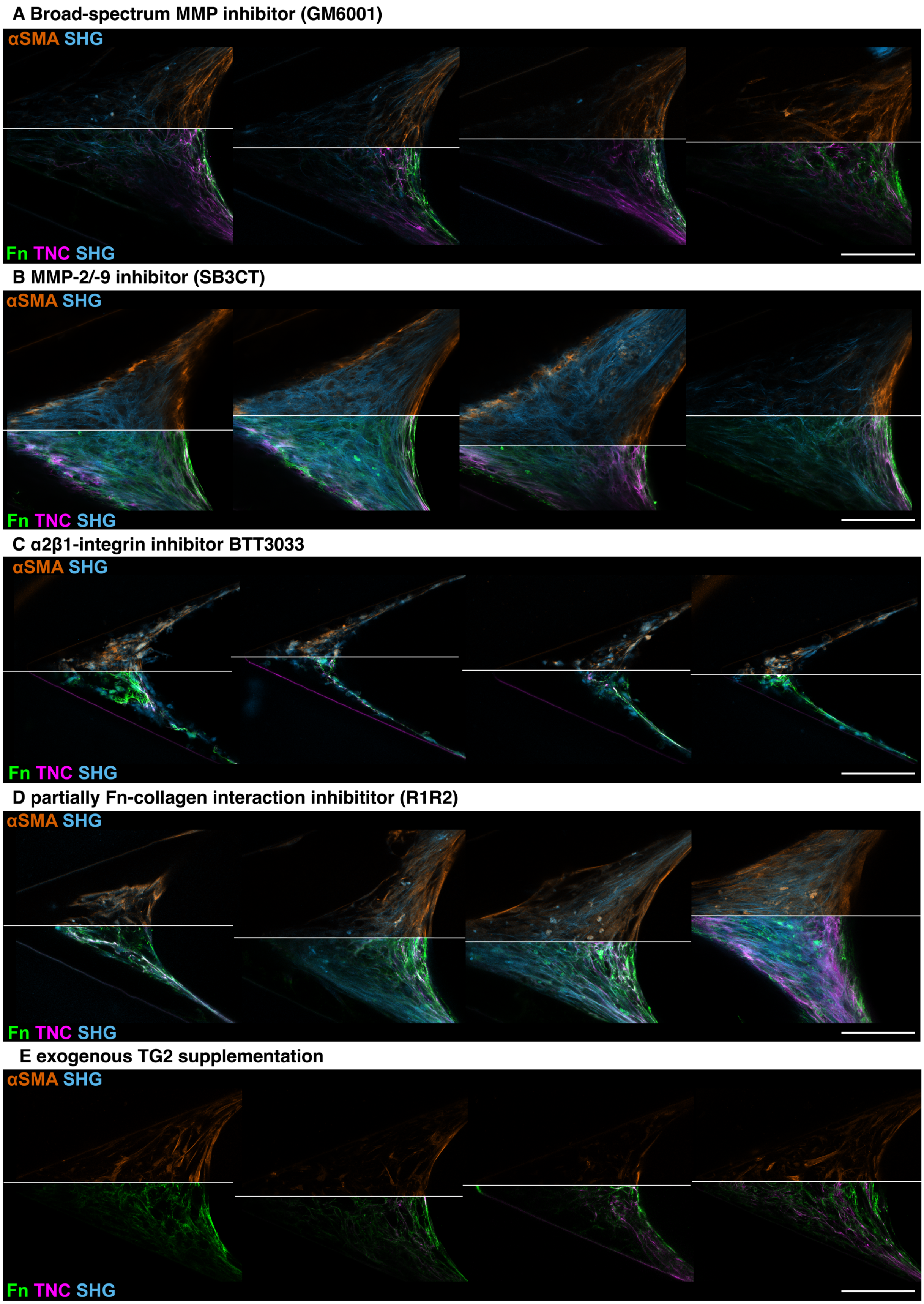
A Broad-spectrum MMP inhibitor (GM6001)
αSMA SHG
Fn TNC SHG
B MMP-2/-9 inhibitor (SB3CT)
αSMA SHG
Fn TNC SHG
C α2β1-integrin inhibitor BTT3033
αSMA SHG
Fn TNC SHG
D partially Fn-collagen interaction inhibititor (R1R2)
αSMA SHG
Fn TNC SHG
E exogenous TG2 supplementation
αSMA SHG
Fn TNC SHG

Fig. S12. Superimposed TNC distribution visualization of four representative µTissues treated with various inhibitors, or upon exogenous TG2 supplementation. The images were taken at the midplane of the µTissues. Data acquired from 12 days old µTissues after interventions with various inhibitors starting at day 4. (**A**-**E**) Superimposed central ROIs of representative µTissues (Sample 1 – 4) treated with various supplements and imaged with confocal and 2-photon laser scanning microscopy (CLSM+SHG) depicted with two different sets of look up tables (top: αSMA (orange), SHG (blue); bottom: Fn (green), TNC (magenta), SHG (blue). Scale bars 100 µm. (**A**) Broad-spectrum MMP inhibition (GM6001, Fig. 4B). (**B**) Specific MMP-2 and 9 inhibitions (SB3CT, Fig. 4C). (**C**) α2β1-integrin inhibition (BTT3033, Fig. 4E). (**D**) Partial inhibition of Fn-collagen interaction (R1R2, Fig. 4D). (**E**) Supplementation of exogenous TG2.

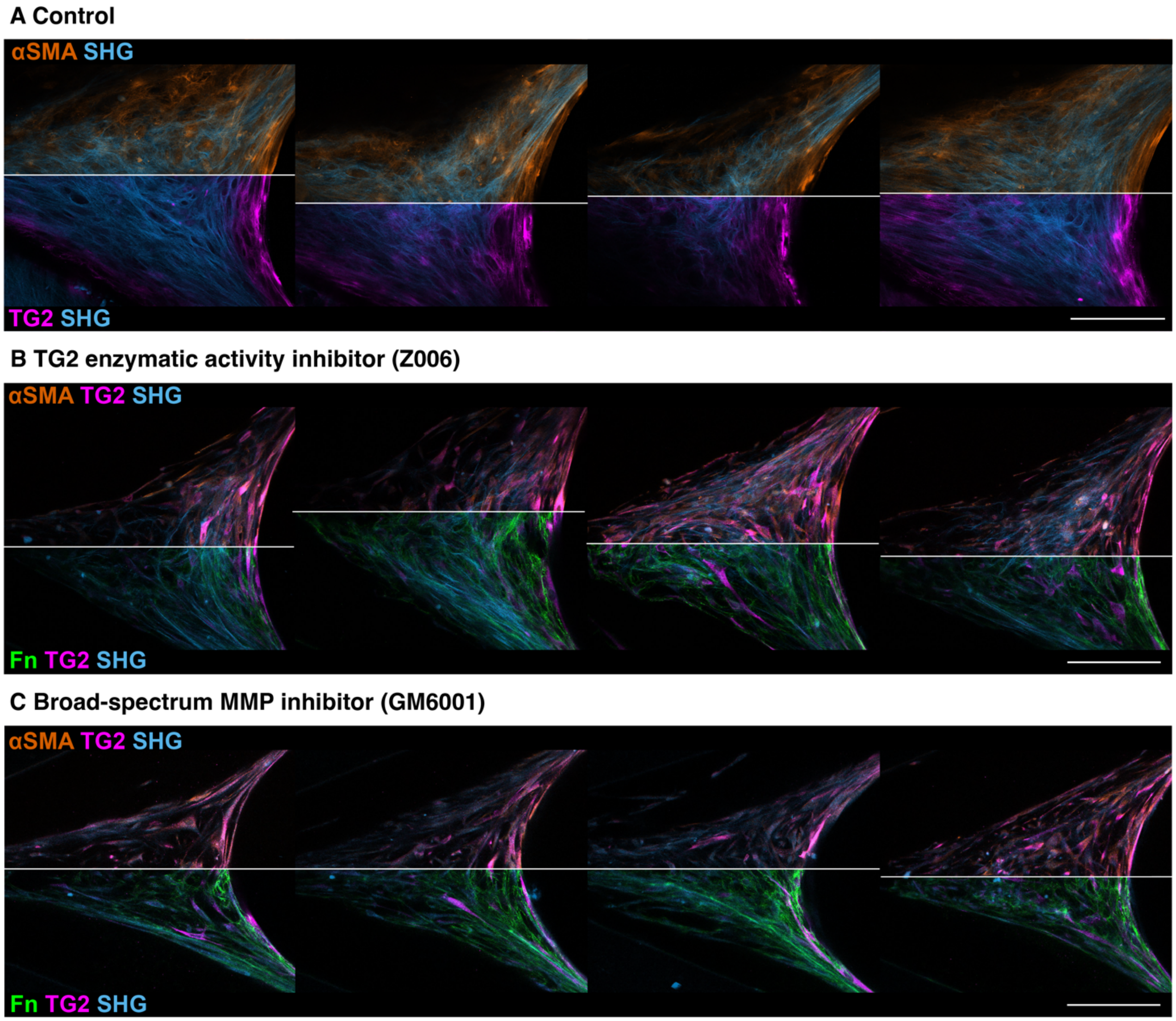

Fig. S13. Superimposed TG2 distribution visualization of various µTissues and various conditions. The images were taken at the midplane of the µTissues. Data acquired from 12 days old µTissues after interventions with various inhibitors starting at day 4. (**A** to **C**) Superimposed central ROIs of representative µTissues (Sample 1 – 4) imaged with confocal and 2-photon laser scanning microscopy (CLSM+SHG) of the (**A**) control (DMSO), (**B**) the specific inhibition of the enzymatic TG2 activity (Z006), and the broad-spectrum MMP inhibition (GM6001). The CLSM+SHG data is depicted with two different sets of look up table; top: αSMA (orange), SHG (cyan); bottom: TG2 (magenta), Fn (green, not available in A), SHG (cyan). Scale bars 100 µm.

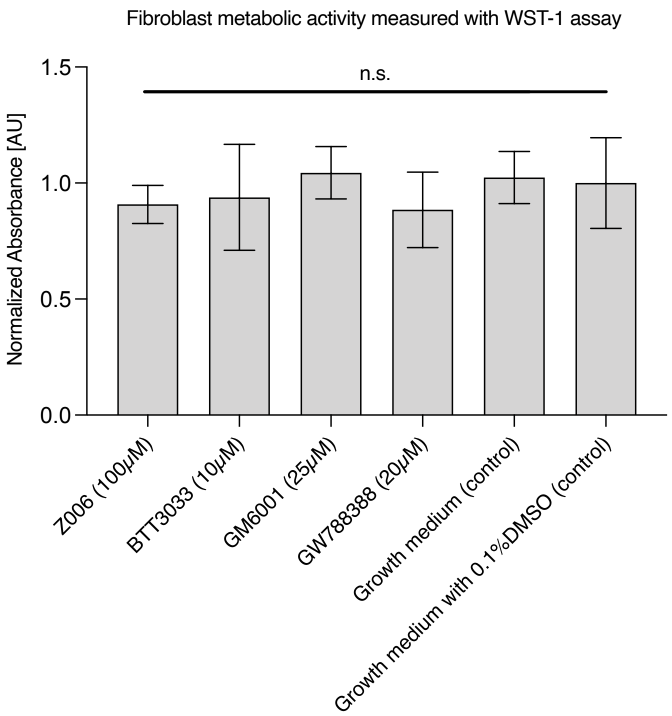

Fig. S14. Fibroblast metabolic activity measured with the WST-1 assay. To assess the potential toxicity of the different inhibitor treatments on cell viability and metabolic activity, a WST-1 test was performed on primary normal dermal human fibroblasts cultured in 2D cell culture. Absorbances values were further normalized to that of the control treated with 0.1% DMSO. After 4 hours, the cells were exposed to one of the following inhibitor treatments: Z006 (100µM, Zedira GmbH), GW788388 (20µM, No 3264, Tocris), GM6001 (25µM, ab120845, Abcam), SB-3CT (5µM, ab141579, Abcam), BTT3033 (10µM, No 4724, Tocris). Cells cultured in normal growth medium and in growth medium supplemented with 0.1% DMSO were used as control, since all inhibitors were dissolved in DMSO. After 24h, tetrazolium salts-containing WST-1 reagent (1:10, No 05015944001, Roche) was added to the cells and incubated for 90 min at 37°C, 5%CO_2. During this time, metabolically active cells cleave the tetrazolium salts contained in the WST-1 reagent into formazan, which has a higher absorbance than the tetrazolium salts. Absorbance values were further normalized to that of the control with 0.1% DMSO supplementation and depicted here. N = 6. Error bars = SD. Statistical significance of the differences between groups was tested using a one-way ANOVA ($p = 0.544$).

Data S1.
Excel spreadsheet containing tabs with data displayed in Figures 1G, 1K+L, 3A-H, 4A-F, 5C.

Movie S1. Sequence of phase-contrast time lapse images from a growing µTissue. The images were taken from day 4 after fibroblast seeding onto the PDMS scaffold for 7 days. Phase-contrast images were taken every 30 min using the cleft array shown in Figure 1. Scale bar 500 µm.

Movie S2. Sequence of phase-contrast time lapse images from a growing and rupturing µTissue treated with R1R2. The images were taken from day 4 after fibroblast seeding onto the PDMS scaffold for 7 days. µTissue was treated with R1R2 supplementation starting on day 4. Phase-contrast images were taken every 30 min. Scale bar 500 µm.
